# Supplementary material for: Application of the ESMO Magnitude of Clinical Benefit Scale to assess the clinical benefit of antibody drug conjugates in solid cancer: a systematic descriptive analysis of phase III and pivotal phase II trials
Source: BMJ Open. 2024 Jun 8;14(6):e077108. doi: 10.1136/bmjopen-2023-077108 (PMC11163648; doi:10.1136/bmjopen-2023-077108)
Supplement: Supplementary data [file bmjopen-2023-077108supp001.pdf]

BMJ Publishing Group Limited (BMJ) disclaims all liability and responsibility arising from any reliance placed on this supplemental material which has been supplied by the author(s)

Supplemental Table S1 Search strategy for studies on ADCs in solid tumours

**Database**	**Search Period**	**Search Terms**
Pubmed	2000 to 18 October 2023	1. antibody-drug conjugates<br>2. solid cancer<br>3. approve<br>4. clinical trials<br>5. quality of life<br>6. follow-up survival<br>7. updated overall survival
