# Supplementary material for: Application of the ESMO Magnitude of Clinical Benefit Scale to assess the clinical benefit of antibody drug conjugates in solid cancer: a systematic descriptive analysis of phase III and pivotal phase II trials
Source: BMJ Open. 2024 Jun 8;14(6):e077108. doi: 10.1136/bmjopen-2023-077108 (PMC11163648; doi:10.1136/bmjopen-2023-077108)
Supplement: Author's manuscript [file bmjopen-2023-077108.draft_revisions.pdf]

BMJ Open is committed to open peer review. As part of this commitment we make the peer review history of every article we publish publicly available.

When an article is published we post the peer reviewers' comments and the authors' responses online. We also post the versions of the paper that were used during peer review. These are the versions that the peer review comments apply to.

The versions of the paper that follow are the versions that were submitted during the peer review process. They are not the versions of record or the final published versions. They should not be cited or distributed as the published version of this manuscript.

BMJ Open is an open access journal and the full, final, typeset and author-corrected version of record of the manuscript is available on our site with no access controls, subscription charges or pay-per-view fees (http://bmjopen.bmj.com).

If you have any questions on BMJ Open's open peer review process please email info.bmjopen@bmj.com

BMJ Open

Application of the ESMO Magnitude of Clinical Benefit Scale to assess the value of antibody–drug conjugates in solid cancer

Journal:	*BMJ Open*
Manuscript ID	bmjopen-2023-077108
Article Type:	Original research
Date Submitted by the Author:	27-Jun-2023
Complete List of Authors:	Ding, Lieming; Betta Pharmaceuticals Co Ltd<br>Yuan, Xiaobin; Betta Pharmaceuticals Co Ltd<br>Wang, Yang; Betta Pharmaceuticals Co Ltd<br>Shen, Zhilin; Betta Pharmaceuticals Co Ltd,<br>Wu, Pengxiang; Betta Pharmaceuticals Co Ltd
Keywords:	ONCOLOGY, PUBLIC HEALTH, Patient-Centered Care

SCHOLARONE™
Manuscripts

I, the Submitting Author has the right to grant and does grant on behalf of all authors of the Work (as defined in the below author licence), an exclusive licence and/or a non-exclusive licence for contributions from authors who are: i) UK Crown employees; ii) where BMJ has agreed a CC-BY licence shall apply, and/or iii) in accordance with the terms applicable for US Federal Government officers or employees acting as part of their official duties; on a worldwide, perpetual, irrevocable, royalty-free basis to BMJ Publishing Group Ltd ("BMJ") its licensees and where the relevant Journal is co-owned by BMJ to the co-owners of the Journal, to publish the Work in this journal and any other BMJ products and to exploit all rights, as set out in our licence.

The Submitting Author accepts and understands that any supply made under these terms is made by BMJ to the Submitting Author unless you are acting as an employee on behalf of your employer or a postgraduate student of an affiliated institution which is paying any applicable article publishing charge ("APC") for Open Access articles. Where the Submitting Author wishes to make the Work available on an Open Access basis (and intends to pay the relevant APC), the terms of reuse of such Open Access shall be governed by a Creative Commons licence – details of these licences and which Creative Commons licence will apply to this Work are set out in our licence referred to above.

Other than as permitted in any relevant BMJ Author's Self Archiving Policies, I confirm this Work has not been accepted for publication elsewhere, is not being considered for publication elsewhere and does not duplicate material already published. I confirm all authors consent to publication of this Work and authorise the granting of this licence.

For peer review only

Application of the ESMO Magnitude of Clinical Benefit Scale to assess the value of antibody–drug conjugates in solid cancer

Running title: Application of the ESMO-MCBS to assess ADCs

Lieming Ding[1,* †], Xiaobin Yuan[1, *], Yang Wang[1], Zhilin Shen[1], Pengxiang Wu[1]

[1]Betta Pharmaceyticals Co., Ltd, Hangzhou, China

[*]These authors contributed equally

[†]Correspondence to: Dr. Lieming Ding. E-mail: lieming.ding@bettapharma.com

For peer review only - http://bmjopen.bmj.com/site/about/guidelines.xhtml

Abstract

Objective: The aim of this study was to assess the clinical benefit value of approved antibody-drug conjugates (ADCs) for solid tumors using the European Society for Medical Oncology Magnitude of Clinical Benefit Scale (ESMO-MCBS) v1.1.

Setting: Descriptive analyses of phase 3 and pivotal phase 2 trials on the ADCs for solid tumors between 2000 and 2023. Palliative treatments were graded 1-5 according to the ESMO-MCBS v1.1, where scores 4 and 5 represent a substantial clinical benefit.

Results: ESMO-MCBS scores were calculated for 19 positive clinical trials. Seven (37%) trials met the ESMO-MCBS benefit threshold grade, while 12 (63%) of the regimens did not meet the ESMO-MCBS benefit threshold grade. Eight of 19 (42%) of the trials had no published scorecard on the ESMO website and discrepancies affected 3 (27%) studies.

Conclusion: More than half of the trials showing a statistically significant result favoring the experimental arm failed to demonstrate a substantial clinical benefit according to the ESMO framework.

For peer review only - http://bmjopen.bmj.com/site/about/guidelines.xhtml

STRENGTHS AND LIMITATIONS OF THIS STUDY

- ESMO-MCBS is a validated tool for grading the clinical benefit of novel drugs.
- To date, few studies evaluate clinical benefit of ADCs therapy, this study firstly assess applicability of ESMO-MCBS in ADCs for solid cancer.
- The data lacks some important information such as the drug costs analysis and ASCO Value Framework, which would provide an improved understanding of the clinical benefit of ADCs.

For peer review only

For peer review only - http://bmjopen.bmj.com/site/about/guidelines.xhtml

Introduction

Cancer is one of the leading causes of the death worldwide, and ranks second in mortality below heart disease in the US.[1,2] Considering the off-target toxicities of chemotherapeutic drugs, novel anti-cancer agents which can selectively target cancer cells and attenuate side effects are urgent. Antibody-drug conjugates (ADCs) are the emerging next generation therapeutics after monoclonal antibodies (mAbs), attribute to the superior anti-cancer activity over traditional chemotherapy.[3,4] To date, a total of 13 ADCs have been approved by Food and Drug Administration (FDA) and more than 100 ADCs are currently being evaluated in clinical trials.[5-8]

The goal of cancer therapy has shifted from the disease-centred strategy to a patient-centred strategy; therefore, the comprehensive value (quality of life [QoL], cost, etc.) of the therapeutic regimen is an increasingly pivotal issue to address. However, it is always hard to objectively evaluate therapy value and clinical benefit. Both the American Society of Clinical Oncology Value Framework (ASCO-VF)[9,10] and the European Society for Medical Oncology Magnitude of Clinical Benefit Scale (ESMO-MCBS)[11,12] aim to assess the clinical benefit of novel cancer therapies. Besides, the ESMO-MCBS seems to be very reliable in advanced or metastatic diseases throughout all treatment settings in daily practice.[13]

Randomized controlled trials are regarded as the 'gold standard' in evaluating medicinal products.[14] Considering an unmet medical need, single-arm trials can also support approvals, although uncommon. Here, we overview the landscape of ADCs approved by FDA, European Medicines Agency (EMA), National Medical Products Administration (NMPA) and Japanese Pharmaceuticals and Medical Devices Agency (PMDA) for solid cancer between 2000 and 2023, and describe the value of these ADCs and further explore whether value is related to the high-quality cancer care.

Methods

Identification and selection of studies

PubMed was searched in May 2023 to identify all the phase 2 and 3 trials in treatment with solid tumors by approved ADCs, by the terms of 'antibody-drug conjugates'

For peer review only - http://bmjopen.bmj.com/site/about/guidelines.xhtml

AND 'phase 2' AND 'phase 3' AND 'cancer'. The single-arm phase 2 trials were included if they were pivotal to obtain its marketing approval.

The exclusion criteria were: non-RCTs and non-pivotal trials (e.g. the secondary, subset, meta-analyses or systematic reviews; phase 1 or 4 trials; exploratory trials); prematurely stopped RCTs; no drug therapy intervention; haematological cancers; non-English articles. The study was reviewed independently by two investigators (XY and YW), and discrepancies were resolved by consensus in the presence of a third investigator (LD).

ESMO-MCBS scoring

The ESMO-MCBS can be used to rank the value of systemic therapies based on reported relative and absolute benefits in terms of improved survival (DFS, PFS and OS) and better survival (e.g. QoL, toxicity). For the non-curative setting reviewed there are two forms (2a and 2b) available that consider the absolute gain in the predefined primary and secondary endpoints and the lower limit of the 95% confidence interval (CI) of the corresponding hazard ratio (HR). For non-inferiority trials, the form 2c was developed and considers QoL/toxicity data for assigning the score. Another form (form 3) is available for the scoring of single-arm studies. The preliminary score was adjusted according to different ESMO stipulations on toxicity, QoL, long-term survival, etc.[15] Palliative treatments were eventually graded 1-5 for advanced disease setting and as A, B, or C for adjuvant or neoadjuvant therapy setting, where scores 5, 4, A or B represent a substantial clinical benefit.

Results

Trial selection and characteristics

A flow chart indicating the selection procedure is shown in figure 1. A total of 25 studies from 19 clinical trials were finally identified, including 6 for trastuzumab emtansine, 1 for enfortumab vedotin, 9 for trastuzumab deruxtecan, 4 for sacituzumab govitecan, 1 for cetuximab saratolacan, 2 for disitamab vedotin, 1 for tisotumab vedotin and 1 for mirvetuximab soravtansine. The approved ADCs covered 7 indications, including breast cancer, urothelial carcinoma, gastric cancer, non-small

For peer review only - http://bmjopen.bmj.com/site/about/guidelines.xhtml

cell lung cancer, head-and-neck squamous cell carcinoma, cervical cancer and ovarian cancer (table 1).[16-40]

ESMO-MCBS scores

In total, 25 publications reporting the results of 19 original clinical trials were assessed. Nine phase 3 studies and 10 phase 2 studies with statistically significant results were graded for clinical benefit using the ESMO-MCBS. 7 (37%) of the regimens met the ESMO-MCBS benefit threshold grade, and 12 (63%) of the regimens did not meet the ESMO-MCBS benefit threshold grade (table 2).

Median PFS and median OS were slightly higher for clinical trials achieving the ESMO threshold for significant clinical benefit (scores 4-5) than for those that did not (scores 1-3). However, the statistically significant differences were not calculated in median PFS and median OS between both groups of trials.

When comparing our final ESMO-MCBS scores with the pertinent ESMO-MCBS scorecards available on the ESMO website, we found that 8/19 (42%) of the trials had no published scorecard; 4 of them on experimental therapies that had granted the market authorization, and continue to be authorized for human use. Discrepancies were observed in 3 of 11 (27%) studies for the following reasons: toxicity and/or QoL adjustment; long-term plateau in the PFS curve; and the selection of ESM0-MCBS v1.1 forms (form 2c vs. form 2a) (table 3).

Discussion

To our knowledge, there is no other review on ESMO-MCBS v1.1 application conducted exclusively in approved ADCs. In the present study, we have applied the ESMO framework to measure the magnitude of clinical benefit into which the results from phases 2 and 3 trials on ADC therapy for solid tumors are translated. Our cohort of 25 studies from 19 clinical trials comprises diverse treatment options with ADCs, from second-line to adjuvant therapies, published between 2000 and 2023.

Phase 3 studies leading to a higher quality of evidence in contrast to phase 2 design. The phase 2 trials included in our study aimed to explore the therapies that granted a market authorization based on preliminary efficacy results. This tendency of

For peer review only - http://bmjopen.bmj.com/site/about/guidelines.xhtml

accelerated authorization from regulatory authorities may contribute to reducing the timeframe for new drugs to enter the market at the expense of clear evidence that they improve patients' OS or QoL, even in post-marketing studies.

The application of MCBS grading forms based on OS and/or PFS was comparable with those based on ORR (42% vs. 53%). In 5 trials with the primary endpoint of PFS, 4 (80%) trials were evaluated with the form 2a instead of form 2b due to the OS advantage. However, a previous study revealed that conclusions based on PFS outcomes in these clinical trials might be overestimated by this scoring system, due to the statistical power to only detect significant differences in PFS, rather than OS.[41] ORR is not a direct measure of clinical benefit; however, it is a measure of anti-cancer activity.[42] A study found that 38% and 34% of anti-cancer drugs were approved by FDA based on ORR and PFS, shortening the development durations by 19 months and 11 months, respectively.[43] Nevertheless, among 93 anti-cancer drug with accelerated approvals, only 20% demonstrated an improvement in OS in confirmatory trials.[44] As previously revealed, the definition of clinical value is different between stakeholders, causing different conclusions.[45] For instance, the ESMO considers benefit as 'living longer and/or living better', which resonates in the ESMO-MCBS form for single-arm trials.[11,12] This is confirmed in our findings that the benefit of the majority of ADCs was 'modest' according to ESMO-MCBS scores. A high ESMO-MCBS score for single-arm trials was related to favorable efficacy in combination with an improved QoL.[46] Yet, delayed publications or publication bias for QoL is overlooked by ESMO-MCBS.[47] Therefore, QoL appears of lesser importance in regulatory decision making on single-arm trials.

Although a minor percentage of discrepancies were found when our scores were compared with the corresponding ESMO scorecards, discrepancies in the value of final scores were major. The re-assessment of the ESMO-MCBS score is important when results from confirmatory trials are published, which may contribute to an improvement in ESMO-MCBS score. Furthermore, extended follow-up may also improve the EMSO-MCBS score. For example, we previously assigned an ESMO-MCBS score of '3' for the sacituzumab govitecan (ASCENT trial) due to the

For peer review only - http://bmjopen.bmj.com/site/about/guidelines.xhtml

increased toxicity; however, we finally assigned a score of '4' due to the a recent publication on health-related QoL.[32] Additionally, adjustments sometimes might be confusing. For example, we assigned an ESMO-MCBS score of '3' for the trastuzumab deruxtecan (DESTINY-Breast03 trial) based on the form 2a due to the improved OS; however, ESMO-MCBS scorecard showed '4' due to the long-term plateau in the PFS curve based on form 2b.

Almost half of positive clinical trials are unable to demonstrate a substantial clinical benefit based on the ESMO framework.[48-50] This issue was criticized after evaluating market approvals for anti-cancer drugs in recent periods of time maximum validity of research data. In our study, only 7/19 (47%) of the clinical trials can meet the ESMO-MCBS threshold for clinical benefit.

Furthermore, we noted that the OS gain in EMILIA was 5.8 months while in DESTINY-Breast02 was 12.7 months in patients with HER2-positive advanced breast cancer, with similar OS in control group and HR.[18,24] The ESMO-MCBS scores were '4' in both trials. Besides, in a head-to-head trial (DESTINY-Breast03) to compare the efficacy and safety of trastuzumab deruxtecan with those of trastuzumab emtansine in patients with HER2-positive metastatic breast cancer, trastuzumab deruxtecan showed a superior OS over trastuzumab emtansine.[26] Thus, we also confused that whether the OS gain in ESMO-MCBS tool can evaluate the benefits of different drugs in the same indication or the same drug in different indications. The thresholds for the OS gain appear a bit lenient.

One of the main limitations is publication selection bias. However, we minimized it by applying an organized searching strategy where two researchers independently selected the studies conforming to predefined inclusion and exclusion criteria. To only assess new therapies that had shown enough efficacy and toxicity data, we discarded phase 1 and non-pivotal phase 2 trials. Thus, the trials included mainly represent commercialized drugs used in daily clinical practice. Besides, the limited toxicity data available within a publication of a clinical trial prevent from properly adjusting the preliminary grades. Additionally, our study was also limited by the lack of drug costs, which may help clinicians to select the optimal therapeutic drugs developed for the

For peer review only - http://bmjopen.bmj.com/site/about/guidelines.xhtml

same clinical entity.[27]

In conclusion, a great proportion of clinical trials on commercialized ADCs cannot meet the ESMO thresholds for meaningful clinical benefit in our study. Despite the ESMO-MCBS v1.1 constitutes a useful and reproducible instrument for assessing the clinical benefit of ADC drugs for solid tumors, a more detailed approach to toxicity/efficacy adjustment is required.

Funding

None.

Contributors

LD, YW and XY designed the study. XY, ZS and PW extracted the data from all sources, performed the analyses and drafted the manuscript. All authors critically revised the manuscript.

Competing interests

Lieming Ding, Xiaobin Yuan, Yang Wang, Zhilin Shen, Pengxiang Wu declare that they are employees of Betta Pharmaceuticals Co., Ltd.

Data availability statement

Data are available upon reasonable request.

For peer review only

References

1. Siegel RL, Miller KD, Fuchs HE, Jemal A. Cancer statistics, 2022. CA Cancer J Clin. 2022;72(1):7-33.

2. Ahmad FB, Anderson RN. The Leading Causes of Death in the US for 2020. JAMA. 2021;325(18):1829-1830.

3. Tarantino P, Carmagnani Pestana R, Corti C, et al. Antibody-drug conjugates: Smart chemotherapy delivery across tumor histologies. CA Cancer J Clin. 2022;72(2):165-182.

4. Chau CH, Steeg PS, Figg WD. Antibody-drug conjugates for cancer. Lancet. 2019;394(10200):793-804.

5. PEG BioPharma (2020) The history of ADC drugs development. https://www.biochempeg.com/article/125.html. Accessed 31 May 2023

6. U.S.F.D.A (2022) FDA D.I.S.C.O. Burst edition: FDA approval of Elahere (mirvetuximab soravtansine-gynx) for FRα positive, platinum-resistant epithelial ovarian, fallopian tube, or peritoneal cancer. https://www.fda.gov/drugs/resources-information-approved-drugs/fda-disco-burst-edition-fda-approval-elahere-mirvetuximab-soravtansine-gynx-fra-positive-platinum. Accessed 31 May 2023

7. Fu C, Yu L, Miao Y, Liu X, Yu Z, Wei M. Peptide-drug conjugates (PDCs): a novel trend of research and development on targeted therapy, hype or hope? Acta Pharm Sin B. 2023;13(2):498-516.

8. Fu Z, Li S, Han S, Shi C, Zhang Y. Antibody drug conjugate: the "biological missile" for targeted cancer therapy. Signal Transduct Target Ther. 2022;7(1):93.

9. Schnipper LE, Davidson NE, Wollins DS, et al. American Society of Clinical Oncology Statement: A Conceptual Framework to Assess the Value of Cancer Treatment Options. J Clin Oncol. 2015;33(23):2563-2577.

10. Schnipper LE, Davidson NE, Wollins DS, et al. Updating the American society of clinical oncology value framework: revisions and reflections in response to comments received. J Clin Oncol. 2016;34(24):2925–2933.

For peer review only - http://bmjopen.bmj.com/site/about/guidelines.xhtml

11. Cherny NI, Sullivan R, Dafni U, et al. A standardised, generic, validated approach to stratify the magnitude of clinical benefit that can be anticipated from anti-cancer therapies: The European Society for Medical Oncology Magnitude of Clinical Benefit Scale (ESMO-MCBS). Ann Oncol. 2015;26(8):1547–1573.

12. Cherny NI, Dafni U, Bogaerts J, et al. ESMO-Magnitude of Clinical Benefit Scale version 1.1. Ann Oncol. 2017;28(10):2340–2366.

13. Kiesewetter B, Raderer M, Steger GG, et al. The European Society for Medical Oncology Magnitude of Clinical Benefit Scale in daily practice: a single institution, real-life experience at the Medical University of Vienna. ESMO Open. 2016;1(4):e000066.

14. Jones D.S., Podolsky S.H. The history and fate of the gold standard. Lancet. 2015;385:1502–1503.

15. Cherny NI, Sullivan R, Dafni U, et al. A standardised, generic, validated approach to stratify the magnitude of clinical benefit that can be anticipated from anti-cancer therapies: the European Society for Medical Oncology Magnitude of Clinical Benefit Scale (ESMO-MCBS). Ann Oncol. 2017;28(11):2901-2905.

16. von Minckwitz G, Huang CS, Mano MS, et al. Trastuzumab Emtansine for Residual Invasive HER2-Positive Breast Cancer. N Engl J Med. 2019;380(7):617-628.

17. Verma S, Miles D, Gianni L, et al. Trastuzumab emtansine for HER2-positive advanced breast cancer. N Engl J Med. 2012;367(19):1783-1791.

18. Diéras V, Miles D, Verma S, et al. Trastuzumab emtansine versus capecitabine plus lapatinib in patients with previously treated HER2-positive advanced breast cancer (EMILIA): a descriptive analysis of final overall survival results from a randomised, open-label, phase 3 trial. Lancet Oncol. 2017;18(6):732-742.

19. Welslau M, Diéras V, Sohn JH, et al. Patient-reported outcomes from EMILIA, a randomized phase 3 study of trastuzumab emtansine (T-DM1) versus capecitabine and lapatinib in human epidermal growth factor receptor 2-positive locally advanced or metastatic breast cancer. Cancer. 2014;120(5):642-651.

For peer review only

For peer review only - http://bmjopen.bmj.com/site/about/guidelines.xhtml

20. Krop IE, Kim SB, González-Martín A, et al. Trastuzumab emtansine versus treatment of physician's choice for pretreated HER2-positive advanced breast cancer (TH3RESA): a randomised, open-label, phase 3 trial. Lancet Oncol. 2014;15(7):689-699.

21. Trastuzumab emtansine versus treatment of physician's choice in patients with previously treated HER2-positive metastatic breast cancer (TH3RESA): final overall survival results from a randomised open-label phase 3 trial. Lancet Oncol. 2017;18(6):743-754.

22. Powles T, Rosenberg JE, Sonpavde GP, et al. Enfortumab Vedotin in Previously Treated Advanced Urothelial Carcinoma. N Engl J Med. 2021;384(12):1125-1135.

23. Modi S, Saura C, Yamashita T, et al. Trastuzumab Deruxtecan in Previously Treated HER2-Positive Breast Cancer. N Engl J Med. 2020;382(7):610-621.

24. André F, Hee Park Y, Kim SB, et al. Trastuzumab deruxtecan versus treatment of physician's choice in patients with HER2-positive metastatic breast cancer (DESTINY-Breast02): a randomised, open-label, multicentre, phase 3 trial. Lancet. 2023;401(10390):1773-1785.

25. Cortés J, Kim SB, Chung WP, et al. Trastuzumab Deruxtecan versus Trastuzumab Emtansine for Breast Cancer. N Engl J Med. 2022;386(12):1143-1154.

26. Hurvitz SA, Hegg R, Chung WP, et al. Trastuzumab deruxtecan versus trastuzumab emtansine in patients with HER2-positive metastatic breast cancer: updated results from DESTINY-Breast03, a randomised, open-label, phase 3 trial. Lancet. 2023;401(10371):105-117.

27. Modi S, Jacot W, Yamashita T, et al. Trastuzumab Deruxtecan in Previously Treated HER2-Low Advanced Breast Cancer. N Engl J Med. 2022;387(1):9-20.

28. Shitara K, Bang YJ, Iwasa S, et al. Trastuzumab Deruxtecan in Previously Treated HER2-Positive Gastric Cancer. N Engl J Med. 2020;382(25):2419-2430.

29. Li BT, Smit EF, Goto Y, et al. Trastuzumab Deruxtecan in HER2-Mutant Non-Small-Cell Lung Cancer. N Engl J Med. 2022;386(3):241-251.

For peer review only - http://bmjopen.bmj.com/site/about/guidelines.xhtml

30. Siena S, Di Bartolomeo M, Raghav K, et al. Trastuzumab deruxtecan (DS-8201) in patients with HER2-expressing metastatic colorectal cancer (DESTINY-CRC01): a multicentre, open-label, phase 2 trial. Lancet Oncol. 2021;22(6):779-789.

31. Yoshino T, Di Bartolomeo M, Raghav K, et al. Final results of DESTINY-CRC01 investigating trastuzumab deruxtecan in patients with HER2-expressing metastatic colorectal cancer. Nat Commun. 2023;14(1):3332.

32. Bardia A, Hurvitz SA, Tolaney SM, et al. Sacituzumab Govitecan in Metastatic Triple-Negative Breast Cancer. N Engl J Med. 2021;384(16):1529-1541.

33. Loibl S, Loirat D, Tolaney SM, et al. Health-related quality of life in the phase III ASCENT trial of sacituzumab govitecan versus standard chemotherapy in metastatic triple-negative breast cancer. Eur J Cancer. 2023;178:23-33.

34. Rugo HS, Bardia A, Marmé F, et al. Sacituzumab Govitecan in Hormone Receptor-Positive/Human Epidermal Growth Factor Receptor 2-Negative Metastatic Breast Cancer. J Clin Oncol. 2022;40(29):3365-3376.

35. Tagawa ST, Balar AV, Petrylak DP, et al. TROPHY-U-01: A Phase II Open-Label Study of Sacituzumab Govitecan in Patients With Metastatic Urothelial Carcinoma Progressing After Platinum-Based Chemotherapy and Checkpoint Inhibitors. J Clin Oncol. 2021;39(22):2474-2485.

36. Cognetti DM, Johnson JM, Curry JM, et al. Phase 1/2a, open-label, multicenter study of RM-1929 photoimmunotherapy in patients with locoregional, recurrent head and neck squamous cell carcinoma. Head Neck. 2021;43(12):3875-3887.

37. Peng Z, Liu T, Wei J, et al. Efficacy and safety of a novel anti-HER2 therapeutic antibody RC48 in patients with HER2-overexpressing, locally advanced or metastatic gastric or gastroesophageal junction cancer: a single-arm phase II study. Cancer Commun (Lond). 2021;41(11):1173-1182.

38. Sheng X, Yan X, Wang L, et al. Open-label, Multicenter, Phase II Study of RC48-ADC, a HER2-Targeting Antibody-Drug Conjugate, in Patients with Locally Advanced or Metastatic Urothelial Carcinoma. Clin Cancer Res. 2021;27(1):43-51.

39. Coleman RL, Lorusso D, Gennigens C, et al. Efficacy and safety of tisotumab vedotin in previously treated recurrent or metastatic cervical cancer (innovaTV

For peer review only - http://bmjopen.bmj.com/site/about/guidelines.xhtml

204/GOG-3023/ENGOT-cx6): a multicentre, open-label, single-arm, phase 2 study. Lancet Oncol. 2021;22(5):609-619.

40. Matulonis UA, Lorusso D, Oaknin A, et al. Efficacy and Safety of Mirvetuximab Soravtansine in Patients With Platinum-Resistant Ovarian Cancer With High Folate Receptor Alpha Expression: Results From the SORAYA Study. J Clin Oncol. 2023;41(13):2436-2445.

41. García-Fumero R, Fernández-López C, Calleja-Hernández MÁ, Expósito-Hernández J. Analyzing the clinical benefit of newer therapies for advanced or metastatic non-small-cell lung cancer: application of the ESMO-magnitude of clinical benefit scale v1.1. Acta Oncol. 2021;60(9):1225-1232.

42. George S.L. Response rate as an endpoint in clinical trial. J Natl Cancer Inst. 2007;99:98–99.

43. Chen EY, Joshi SK, Tran A, Prasad V. Estimation of Study Time Reduction Using Surrogate End Points Rather Than Overall Survival in Oncology Clinical Trials. JAMA Intern Med. 2019;179(5):642-647.

44. Gyawali B, Hey SP, Kesselheim AS. Assessment of the clinical benefit of cancer drugs receiving accelerated approval. JAMA Intern Med. 2019;179(7):906-913.

45. Pignatti F, Wilking U, Postmus D, et al. The value of anticancer drugs-a regulatory view. Nat Rev Clin Oncol. 2022;19:207–215.

46. Tibau A, Molto C, Borrell M, et al. Magnitude of Clinical Benefit of Cancer Drugs Approved by the US Food and Drug Administration Based on Single-Arm Trials. JAMA Oncol. 2018;4(11):1610-1611.

47. Gyawali B, de Vries EGE, Dafni U, et al. Biases in study design, implementation, and data analysis that distort the appraisal of clinical benefit and ESMO-Magnitude of Clinical Benefit Scale (ESMO-MCBS) scoring. ESMO Open. 2021;6(3):100117.

48. Davis C, Naci H, Gurpinar E, Poplavska E, Pinto A, Aggarwal A. Availability of evidence of benefits on overall survival and quality of life of cancer drugs approved by European Medicines Agency: retrospective cohort study of drug approvals 2009-13. BMJ. 2017;359:j4530.

For peer review only - http://bmjopen.bmj.com/site/about/guidelines.xhtml

49. Naci H, Davis C, Savović J, et al. Design characteristics, risk of bias, and reporting of randomised controlled trials supporting approvals of cancer drugs by European Medicines Agency, 2014-16: cross sectional analysis. BMJ. 2019;366:l5221.

50. Hilal T, Gonzalez-Velez M, Prasad V. Limitations in Clinical Trials Leading to Anticancer Drug Approvals by the US Food and Drug Administration. JAMA Intern Med. 2020;180(8):1108-1115.

51. Hammerman A, Greenberg-Dotan S, Feldhamer I, Birnbaum Y, Cherny NI. The ESMO-Magnitude of Clinical Benefit Scale for novel oncology drugs: correspondence with three years of reimbursement decisions in Israel. Expert Rev Pharmacoecon Outcomes Res. 2018;18(1):119-122.

For peer review only

Figure 1 Flow diagram

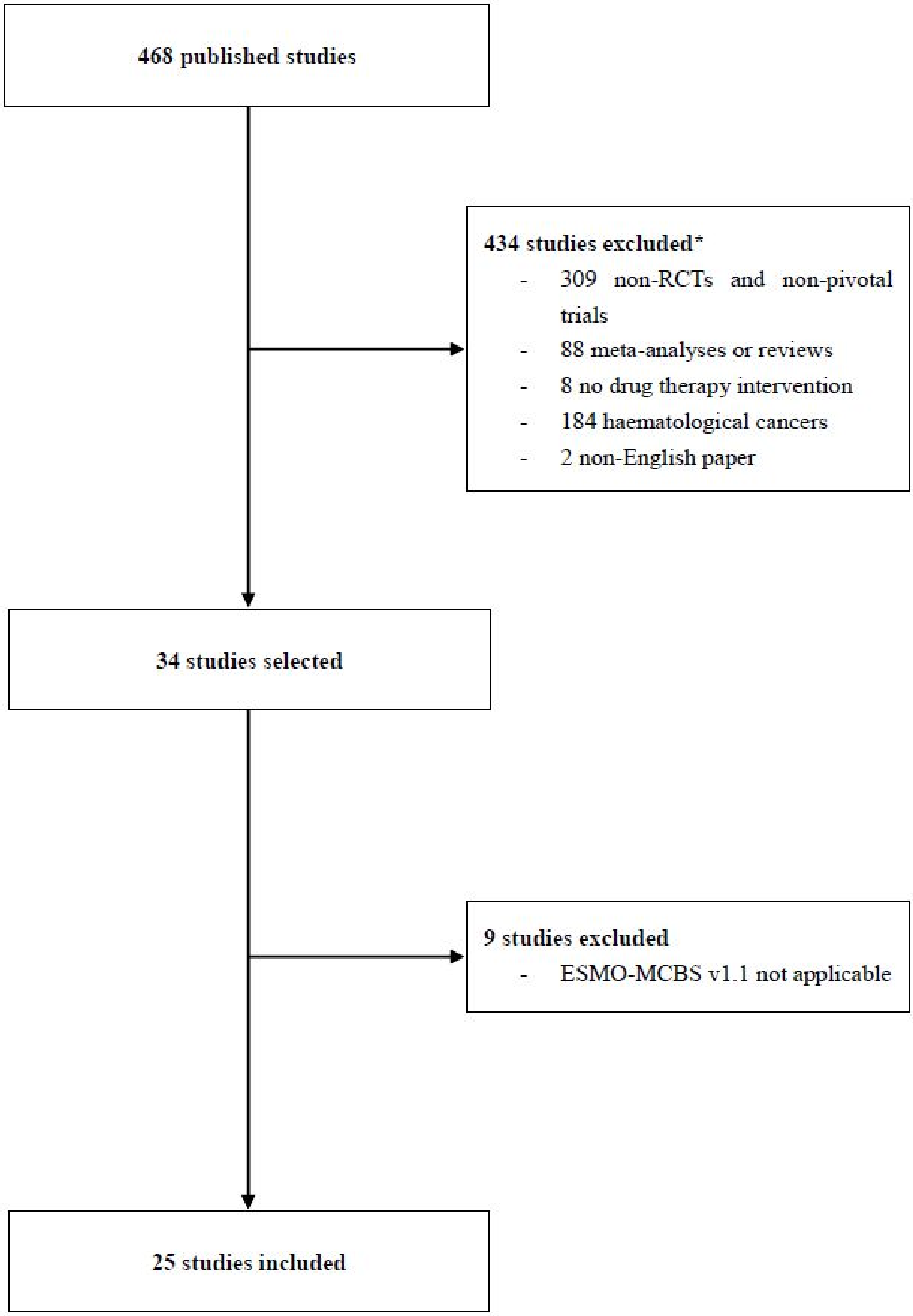

*some have multiple exclusion criteria

For peer review only - http://bmjopen.bmj.com/site/about/guidelines.xhtml

Table 1 Characteristics of the included studies

Drug name	Trial name	Registry number	Tumour type	Approval	Ref
Trastuzumab emtansine (T-DM1)	KATHERINE	NCT01772472	HER2-positive early breast cancer who had residual invasive disease in the breast or axilla at surgery after receiving neoadjuvant therapy containing a taxane (with or without anthracycline) and trastuzumab	Yes	16
	EMILIA	NCT00829166	HER2-positive advanced breast cancer second line	Yes	17-19
	TH3RESA	NCT01419197	HER2-positive advanced breast cancer	-	20,21
Enfortumab vedotin	EV-301	NCT03474107	Locally advanced or metastatic urothelial carcinoma	Yes	22
Trastuzumab deruxtecan (DS-8201)	DESTINY-Breast01	NCT03248492	HER2-positive metastatic breast cancer third line	Yes	23
	DESTINY-Breast02	NCT03523585	HER2-positive metastatic breast cancer third line	Yes	24
	DESTINY-Breast03	NCT03529110	HER2-positive metastatic breast cancer second line	Yes	25,26
	DESTINY-Breast04	NCT03734029	HER2-low metastatic breast cancer	Yes	27
	DESTINY-Gastric01	NCT03329690	HER2-positive advanced gastric cancer	Yes	28
	DESTINY-Lung01	NCT03505710	metastatic HER2-mutant NSCLC second line	Yes	29
	DESTINY-CRC01	NCT03384940	HER2-positive metastatic colorectal cancer	No	30,31
Sacituzumab govitecan	ASCENT	NCT02574455	Relapsed or refractory metastatic triple-negative breast cancer	Yes	32,33
	TROPiCS-02	NCT03901339	Endocrine-resistant, chemotherapy- treated HR+/HER2- locally recurrent inoperable or metastatic breast cancer	Yes	34
	TROPHY-U-01	NCT03547973	Metastatic urothelial carcinoma	Yes	35
Cetuximab saratolacan	-	NCT02422979	Recurrent head and neck squamous cell carcinoma	Yes	36
Disitamab vedotin	-	NCT03556345	HER2-overexpressing, locally advanced or metastatic gastric or gastroesophageal junction cancer	Yes	37
	-	NCT03507166	HER2+ locally advanced or metastatic urothelial carcinoma	Yes	38
Tisotumab vedotin	innovaTV 204	NCT03438396	Recurrent or metastatic cervical cancer	Yes	39
Mirvetuximab soravtansine	SORAYA	NCT04296890	FRa-high, platinum-resistant epithelial ovarian cancer	Yes	40

For peer review only - http://bmjopen.bmj.com/site/about/guidelines.xhtml

Table 2 Clinical benefit according to ESMO-MCBS v1.1

Trial name	Intervention group	Primary outcome (PO)	PO control group	PO gain	PO HR (95% CI)	Toxicity/QoL	ESM0-MCBS v1.1
KATHERINE	T-DM1	iDFS	3-year: 77.0%	3-year: 11.3%	0.50 (0.39-0.64)	NA	A (form 1)
EMILIA	T-DM1	PFS and OS	25.1 mo	5.8 mo	0.68 (0.55-0.85)	NA	4 (form 2a)
TH3RESA	T-DM1	PFS and OS	15.8 mo	6.9 mo	0.68 (0.54-0.85)	NA	4 (form 2a)
EV-301	Enfortumab vedotin	OS	8.97 mo	3.91 mo	0.70 (0.56-0.89)	NA	4 (form 2a)
DESTINY-Breast01*	DS-8201	ORR	-	-	-	NA	3 (form 3)
DESTINY-Breast02	DS-8201	PFS (OS improved)	26.5 mo	12.7 mo	0.66 (0.50-0.86)	NA	4 (form 2a)
DESTINY-Breast03	DS-8201	PFS (OS improved)	2-year: 69.9%	2-year: 7.5%	0.64 (0.47-0.87)	NA	3 (form 2a)
DESTINY-Breast04	DS-8201	PFS (OS improved)	16.8 mo	6.6 mo	0.64 (0.49–0.84)	NA	4 (form 2a)
DESTINY-Gastric01#	DS-8201	ORR	14%	37%	-	NA	2 (form 2c)
DESTINY-Lung01*	DS-8201	ORR	-	-	-	NA	3 (form 3)
DESTINY-CRC01*	DS-8201	ORR	-	-	-	NA	3 (form 3)
ASCENT	Sacituzumab govitecan	PFS (OS improved)	6.9 mo	4.9 mo	0.51 (0.41-0.62)	Increased toxicity/ Improved HRQoL	4 (form 2a)
TROPiCS-02	Sacituzumab govitecan	PFS	4.0 mo	1.5 mo	0.66 (0.53-0.83)	NA	3 (form 2b)
TROPHY-U-01*	Sacituzumab govitecan	ORR	-	-	-	NA	3 (form 3)
NCT02422979*	Cetuximab saratolacan	ORR	-	-	-	NA	2 (form 3)
NCT03556345*	Disitamab vedotin	ORR	-	-	-	NA	2 (form 3)
NCT03507166*	Disitamab vedotin	ORR	-	-	-	NA	3 (form 3)
innovaTV 204*	Tisotumab vedotin	ORR	-	-	-	NA	2 (form 3)
SORAYA*	Mirvetuximab soravtansine	ORR	-	-	-	NA	2 (form 3)

*Single-arm, phase 2 trial

#Randomized, phase 2 trial

For peer review only

For peer review only - http://bmjopen.bmj.com/site/about/guidelines.xhtml

Table 3 Discrepancies between ESMO framework in this study and the ESMO-MCBS scorecards

Trial name	ESM0-MCBS v1.1	ESMO-MCBS scorecards	Reason for the discrepancies
KATHERINE	A (form 1)	A	NA
EMILIA	4 (form 2a)	4	NA
EV-301	4 (form 2a)	4	NA
DESTINY-Breast01	3 (form 3)	2	Toxicity adjustment
DESTINY-Breast03	3 (form 2a)	4	Long-term plateau in the PFS curve
DESTINY-Breast04	4 (form 2a)	4	NA
DESTINY-Gastric01	2 (form 2c)	4	Selection of ESM0-MCBS v1.1 forms
ASCENT	4 (form 2a)	4	NA
TROPiCS-02	3 (form 2b)	3	NA
innovaTV 204	2 (form 3)	2	NA
SORAYA	2 (form 3)	2	NA

For peer review only

For peer review only - http://bmjopen.bmj.com/site/about/guidelines.xhtml

BMJ Open

Application of the ESMO Magnitude of Clinical Benefit Scale to assess the clinical benefit of antibody–drug conjugates in solid cancer: a systematic descriptive analysis of phase 3 and pivotal phase 2 trials

Journal:	*BMJ Open*
Manuscript ID	bmjopen-2023-077108.R1
Article Type:	Original research
Date Submitted by the Author:	24-Oct-2023
Complete List of Authors:	Ding, Lieming; Betta Pharmaceuticals Co Ltd<br>Yuan, Xiaobin; Betta Pharmaceuticals Co Ltd<br>Wang, Yang; Betta Pharmaceuticals Co Ltd<br>Shen, Zhilin; Betta Pharmaceuticals Co Ltd,<br>Wu, Pengxiang; Betta Pharmaceuticals Co Ltd
**Primary Subject Heading**:	Oncology
Secondary Subject Heading:	Global health
Keywords:	ONCOLOGY, PUBLIC HEALTH, Patient-Centered Care

SCHOLARONE™
Manuscripts

I, the Submitting Author has the right to grant and does grant on behalf of all authors of the Work (as defined in the below author licence), an exclusive licence and/or a non-exclusive licence for contributions from authors who are: i) UK Crown employees; ii) where BMJ has agreed a CC-BY licence shall apply, and/or iii) in accordance with the terms applicable for US Federal Government officers or employees acting as part of their official duties; on a worldwide, perpetual, irrevocable, royalty-free basis to BMJ Publishing Group Ltd ("BMJ") its licensees and where the relevant Journal is co-owned by BMJ to the co-owners of the Journal, to publish the Work in this journal and any other BMJ products and to exploit all rights, as set out in our licence.

The Submitting Author accepts and understands that any supply made under these terms is made by BMJ to the Submitting Author unless you are acting as an employee on behalf of your employer or a postgraduate student of an affiliated institution which is paying any applicable article publishing charge ("APC") for Open Access articles. Where the Submitting Author wishes to make the Work available on an Open Access basis (and intends to pay the relevant APC), the terms of reuse of such Open Access shall be governed by a Creative Commons licence – details of these licences and which Creative Commons licence will apply to this Work are set out in our licence referred to above.

Other than as permitted in any relevant BMJ Author's Self Archiving Policies, I confirm this Work has not been accepted for publication elsewhere, is not being considered for publication elsewhere and does not duplicate material already published. I confirm all authors consent to publication of this Work and authorise the granting of this licence.

For peer review only

Application of the ESMO Magnitude of Clinical Benefit Scale to assess the clinical benefit of antibody–drug conjugates in solid cancer: a systematic descriptive analysis of phase 3 and pivotal phase 2 trials

Running title: Application of the ESMO-MCBS to assess ADCs

Lieming Ding[1,*,†], Xiaobin Yuan[1,*], Yang Wang[1], Zhilin Shen[1], Pengxiang Wu[1]

[1]Betta Pharmaceuticals Co., Ltd, Hangzhou, China

[*]These authors contributed equally

[†]Correspondence to: Dr. Lieming Ding. E-mail: lieming.ding@bettapharma.com

For peer review only - http://bmjopen.bmj.com/site/about/guidelines.xhtml

Abstract

Objective: The aim of this study was to assess the clinical benefit value of approved antibody-drug conjugates (ADCs) for solid tumors using the European Society for Medical Oncology Magnitude of Clinical Benefit Scale (ESMO-MCBS) v1.1.

Design: A systematic descriptive analysis between 2000 and 2023 per the ESMO-MCBS v1.1.

Data sources: PubMed was searched by 18 October 2023.

Eligibility criteria: We included the phase 3 randomised controlled trials or phase 2 pivotal trials leading to approval of ADCs in solid tumors.

Data extraction and synthesis: Two independent reviewers extracted data and discrepancies were resolved by consensus in the presence of a third investigator.

Results: ESMO-MCBS scores were calculated for 17 positive clinical trials on 8 ADCs, which were firstly approved by US Food and Drug Administration (FDA), European Medicines Agency (EMA), China National Medical Products Administration (NMPA) and Japanese Pharmaceuticals and Medical Devices Agency (PMDA) for solid cancers. Among 17 trials, 4 (24%) trials met the ESMO-MCBS benefit threshold grade, while 13 (76%) of the regimens did not meet the ESMO-MCBS benefit threshold grade. Eight of 19 (42%) of the trials had no published scorecard on the ESMO website due to the approval by other jurisdictions not by FDA or EMA. The discrepancies between our results and ESMO scorecard were observed in 5/12 (42%) trials, mostly owing to integration of more recent data.

Conclusions: ESMO-MCBS is an important tool for assessing clinical benefit of cancer drugs, although not all drugs met the meaningful threshold. Our study suggest that subsequent clinical trials on the treatment of solid tumors with ADCs should follow the cases with meaningful clinical benefit..

For peer review only - http://bmjopen.bmj.com/site/about/guidelines.xhtml

STRENGTHS AND LIMITATIONS OF THIS STUDY

- This analysis assessed clinical benefit of either phase 3 randomised controlled trials or phase 2 pivotal trials leading to approval.
- ESMO-MCBS is a validated tool for grading the clinical benefit of novel drugs.
- Shortcomings of ESMO-MCBS were identified and need to address in the future version.
- The data lacks some important information such as the drug costs analysis and ASCO Value Framework.

For peer review only

For peer review only - http://bmjopen.bmj.com/site/about/guidelines.xhtml

Introduction

Cancer is one of the leading causes of the death worldwide, and ranks second in mortality below heart disease in the US [1, 2].Considering the off-target toxicities of chemotherapeutic drugs, novel anti-cancer agents which can selectively target cancer cells and attenuate side effects are urgent. Antibody-drug conjugates (ADCs) are the emerging next generation therapeutics after monoclonal antibodies (mAbs), attribute to the superior anti-cancer activity over traditional chemotherapy [3, 4].In 2000, US Food and Drug Administration (FDA) approved the first ADC, gemtuzumab ozogamicin (Mylotarg), for the treatment of patients with acute myeloid leukemia (AML) [5]. To date, a total of 15 ADCs have been approved worldwide and more than 100 ADCs are currently being evaluated in clinical trials [6].

The goal of cancer therapy has shifted from the disease-centred strategy to a patient-centred strategy; therefore, the comprehensive value (quality of life [QoL], cost, etc.) of the therapeutic regimen is an increasingly pivotal issue to address. However, it is always hard to objectively evaluate therapy value and clinical benefit. Both the American Society of Clinical Oncology Value Framework (ASCO-VF) [7, 8] and the European Society for Medical Oncology Magnitude of Clinical Benefit Scale (ESMO-MCBS) [9, 10] aim to assess the clinical benefit of novel cancer therapies. Besides, the ESMO-MCBS seems to be very reliable in advanced or metastatic diseases throughout all treatment settings in daily practice [11].

Randomized controlled trials are regarded as the 'gold standard' in evaluating medicinal products [12]. Considering an unmet medical need, single-arm trials can also support approvals, although uncommon. Here, we overview the landscape of ADCs approved by FDA, European Medicines Agency (EMA), China National Medical Products Administration (NMPA) and Japanese Pharmaceuticals and Medical Devices Agency (PMDA) for solid cancer, and describe the clinical benefit of these ADCs and further explore whether clinical benefit is related to the high-quality cancer care.

Methods

For peer review only - http://bmjopen.bmj.com/site/about/guidelines.xhtml

Design

Our study is a systematic descriptive analysis without protocol and was not registered.

Data sources and searches

PubMed (https://pubmed.ncbi.nlm.nih.gov/) was searched in from 2000 to 18 October, 2023. The full search terms used in this study are presented in supplementary table S1. The search was limited to human studies and had no language restrictions.

Study selection

The inclusion criterions were the phase 2 and phase 3 randomized controlled trials that led to approval by regulatory authorities. The phase 2 single-arm trials were included if they were pivotal to obtain its marketing approval. Updated data of pivotal studies or reports on toxicity or QoL outcomes were also considered.

The exclusion criteria were: non-pivotal trials (e.g. the secondary, subset, meta-analyses or systematic reviews; phase 1 or 4 trials; exploratory trials); prematurely stopped RCTs; no drug therapy intervention; haematological cancers; non-English articles.

Data extraction

The study was reviewed independently by two investigators (XY and YW) and extracted relevant details from each study, including study design, sample size and endpoints (objective response rate [ORR], disease-free survival [DFS], progression free survival [PFS], overall survival [OS], QoL and toxicity). The discrepancies were resolved by consensus in the presence of a third investigator (LD).

ESMO-MCBS scoring

The ESMO-MCBS can be used to rank the value of systemic therapies based on reported relative and absolute benefits in terms of improved survival (DFS, PFS and OS) and better survival (e.g. QoL, toxicity). It is worth noting that the ESMO-MCBS can only be applied to studies showing statistically significant differences. For the non-curative setting reviewed there are two forms (2a and 2b) available that consider the absolute gain in the predefined primary and secondary endpoints and the lower limit of the 95% confidence interval (CI) of the corresponding hazard ratio (HR). For non-inferiority trials, the form 2c was developed and considers QoL/toxicity data for

For peer review only - http://bmjopen.bmj.com/site/about/guidelines.xhtml

assigning the score. Another form (form 3) is available for the scoring of single-arm studies. The preliminary score was adjusted according to different ESMO stipulations on toxicity, QoL, long-term survival, etc [13]. Palliative treatments were eventually graded 1-5 for advanced disease setting and as A, B, or C for adjuvant or neoadjuvant therapy setting, where scores 5, 4, A or B represent a substantial clinical benefit.

Patient and public involvement

No patients were directly involved in the development of the research question, selection of the outcome measures, design and implementation of the study, or interpretation of the results.

Results

Trial selection and characteristics

After excluding 7 of 15 approved ADCs (i.e. gemtuzumab ozogamicin, brentuximab vedotin, inotuzumab ozogamicin, moxetumomab pasudotox, polatuzumab vedotin, belantamab mafodotin and loncastuximab tesirine) for haematological cancers, clinical trials on 8 ADCs for solid cancers were identified. A total of 22 publications from 17 clinical trials (9 randomized controlled trials and 8 single-arm trials) were finally identified, including 4 for trastuzumab emtansine, 1 for enfortumab vedotin, 7 for trastuzumab deruxtecan, 5 for sacituzumab govitecan, 1 for cetuximab saratolacan, 2 for disitamab vedotin, 1 for tisotumab vedotin and 1 for mirvetuximab soravtansine. The approved ADCs covered 7 cancer types, including breast cancer, urothelial carcinoma, gastric cancer, non-small cell lung cancer, head-and-neck squamous cell carcinoma, cervical cancer and ovarian cancer (table 1) [14-35].

ESMO-MCBS scores

In total, 17 clinical trials were assessed by form 1 (n = 1), form 2a (n = 5), form 2b (n = 1), form 2c (n = 1) and form 3 (n = 9). Among 17 trials, 4 (24%) of the regimens met the ESMO-MCBS benefit threshold grade, while 13 (76%) of the regimens did not meet the ESMO-MCBS benefit threshold grade (table 2).

Median PFS and median OS were slightly higher for clinical trials achieving the ESMO threshold for significant clinical benefit (scores 4-5) than for those that did not

For peer review only - http://bmjopen.bmj.com/site/about/guidelines.xhtml

(scores 1-3). However, the statistically significant differences were not calculated in median PFS and median OS between both groups of trials.

When comparing our final ESMO-MCBS scores with the pertinent ESMO-MCBS scorecards available on the ESMO website, we found that 5/17 (29%) of the trials had no published scorecard, mostly because they are approved by jurisdictions other than the EMA and FDA. We think this may not contribute to adequate education and promotion worldwide. In our study, several ADCs have been approved by local agencies in China and Japan. We suggest that ESMO-MCBS scorecards can also published the drugs that are approved by other agencies other than FDA and EMA. Discrepancies were observed in 5 of 12 (42%) trials (table 2). We find different scores mostly due to integration of more recent data. When comes to multiple publications, the clinical benefit was evaluated based on both the primary and secondary manuscripts. While we were always presented the scores according to the newly published articles due to the more mature OS data or QoL data. Interestingly, discrepancies were observed in EMILIA and TROPiCS trials. The scores in EMILIA based on the interim analyses and final OS analyses were 4 and 3, respectively. The score was decreased as the OS HR was increased. Similarly, the scores in TROPiCS-02 based on the interim analyses and final OS analyses were 3 and 1, respectively. As OS data was mature and showed benefit, the form 2b was replaced by form 2a; however, the OS HR was increased, leading the low score.

Discussion

To our knowledge, there is no other review on ESMO-MCBS v1.1 application conducted exclusively in approved ADCs. In the present study, we have applied the ESMO framework to measure the magnitude of clinical benefit into which the results from phases 2 and 3 trials on ADC therapy for solid tumors are translated. Our cohort of 22 publications from 17 clinical trials comprises diverse treatment options with ADCs, from second-line to adjuvant therapies, published between 2000 and 2023.

Phase 3 design leading to a higher quality of evidence in contrast to phase 2 design. The phase 2 trials included in our study aimed to explore the therapies that granted a

For peer review only - http://bmjopen.bmj.com/site/about/guidelines.xhtml

market authorization based on preliminary efficacy results. This tendency of accelerated authorization from regulatory authorities may contribute to reducing the timeframe for new drugs to enter the market at the expense of clear evidence that they improve patients' OS or QoL, even in post-marketing studies.

The application of MCBS grading forms based on OS and/or PFS was less than those based on ORR (35% vs. 59%). In 5 trials with the primary endpoint of PFS, 4 (80%) trials were evaluated with the form 2a instead of form 2b due to the OS advantage. However, a previous study revealed that conclusions based on PFS outcomes in these clinical trials might be overestimated by this scoring system, due to the statistical power to only detect significant differences in PFS, rather than OS [36]. ORR is not a direct measure of clinical benefit; however, it is a measure of anti-cancer activity [37]. A study found that 38% and 34% of anti-cancer drugs were approved by FDA based on ORR and PFS, shortening the development durations by 19 months and 11 months, respectively [38]. Nevertheless, among 93 anti-cancer drug with accelerated approvals, only 20% demonstrated an improvement in OS in confirmatory trials [39]. As previously revealed, the definition of clinical value is different between stakeholders, causing different conclusions [40]. For instance, the ESMO considers benefit as 'living longer and/or living better', which resonates in the ESMO-MCBS form for single-arm trials [9, 10]. This is confirmed in our findings that the benefit of the majority of ADCs was 'modest' according to ESMO-MCBS scores. A high ESMO-MCBS score for single-arm trials was related to favorable efficacy in combination with an improved QoL [41]. Yet, delayed publications or publication bias for QoL is overlooked by ESMO-MCBS [42]. Therefore, QoL appears of lesser importance in regulatory decision making on single-arm trials.

Although a minor percentage of discrepancies were found when our scores were compared with the corresponding ESMO scorecards, discrepancies in the value of final scores were major. The re-assessment of the ESMO-MCBS score is important when results from confirmatory trials are published, which may affect ESMO-MCBS score. Furthermore, extended follow-up may also affect the EMSO-MCBS score. For example, we previously assigned an ESMO-MCBS score of '3' for the sacituzumab

For peer review only - http://bmjopen.bmj.com/site/about/guidelines.xhtml

govitecan (ASCENT trial) due to the increased toxicity; however, we finally assigned a score of '4' due to the a recent publication on health-related QoL [26]. On the contrary, the score was found to decrease as OS data was mature and showed benefit while the value of OS HR was increased in EMILIA [15, 16] and TROPiCS trials [28, 29]. ESMO-MCBS can develop specialized form for re-evaluation of the updated OS data. Additionally, adjustments sometimes might be confusing. For example, we assigned an ESMO-MCBS score of '3' for the trastuzumab deruxtecan (DESTINY-Breast03 trial) based on the form 2a due to the improved OS; however, ESMO-MCBS scorecard showed '4' due to the long-term plateau in the PFS curve based on form 2b. Although grades 3 or higher adverse events differ between treatment arms, the statistical significance is always not available in the publications. Besides, any adverse events of grade 3 or higher but not those affecting patients' daily well-being, as denoted in the ESMO-MCBS v1.1 forms, were published. Further versions of the ESMO-MCBS that can clearly defined or quantified toxicity profiles are highly expected and desirable.

Almost half of positive clinical trials are unable to demonstrate a substantial clinical benefit based on the ESMO framework [43-45]. This issue was criticized after evaluating market approvals for anti-cancer drugs in recent periods of time maximum validity of research data. In our study, only 4/17 (24%) of the clinical trials can meet the ESMO-MCBS threshold for clinical benefit. Notably, all single-arm trial cannot meet the ESMO-MCBS threshold for clinical benefit. Form 3 is used to assess single-arm trials, but it should also be noted that by utilizing this form, only grades from 1 to 4 can be attained. As a result, the threshold can only be met if the study demonstrates HRQoL improvements, or additionally, if confirmatory phase 4 trials are accessible.

Furthermore, in a head-to-head trial (DESTINY-Breast03) to compare the efficacy and safety of trastuzumab deruxtecan with those of trastuzumab emtansine in patients with HER2-positive metastatic breast cancer, trastuzumab deruxtecan showed a superior OS over trastuzumab emtansine [21]. Thus, we also confused that whether the OS gain in ESMO-MCBS tool can evaluate the benefits of different drugs in the

For peer review only - http://bmjopen.bmj.com/site/about/guidelines.xhtml

same indication or the same drug in different indications. The thresholds for the OS gain appear a bit lenient.

This study has several implications. Firstly, this study showed clinical benefits for limited ADCs in solid tumors, suggesting that subsequent clinical trials on the treatment of solid tumors with ADCs should follow the cases with meaningful clinical benefit. Secondly, value frameworks can help not only identify drugs with low or uncertain clinical benefit that should be targeted for price negotiations, but also therapies with evidence of higher clinical benefit to improve access to benefit drugs, thereby contributing to patient-centred cancer treatment goals. Finally, the ADCs with HRQoL improvement showed clinical benefit, suggesting that HRQoL should be paid sufficient attention in clinical trials and clinical treatment strategies.

There are several limitations in our study. First, we limited our analysis to solid tumors and excluded drugs approved to treat hematologic malignancies. When this study was completed in June 2023, the haematology-specific version has yet to be published. We will further access the clinical benefits of ADCs for hematologic malignancies according to the currently published ESMO-MCBS:H v 1.0 [46]. Another main limitations is publication selection bias. However, we minimized it by applying an organized searching strategy where two researchers independently selected the studies conforming to predefined inclusion and exclusion criteria. To only assess new therapies that had shown enough efficacy and toxicity data, we discarded phase 1 and non-pivotal phase 2 trials. Thus, the trials included mainly represent commercialized drugs used in daily clinical practice. Besides, the limited toxicity data available within a publication of a clinical trial prevent from properly adjusting the preliminary grades. Additionally, our study was also limited by the lack of drug costs, which may help clinicians to select the optimal therapeutic drugs developed for the same clinical entity [22].

In conclusion, this is the first study that used ESMO-MCBS tool to assess clinical benefit of ADCs across several solid cancers. ESMO-MCBS are important tools for assessing clinical benefit of cancer drugs, although not all drugs met the meaningful threshold. Those therapeutic regimens with improved HRQoL showed clinical value,

For peer review only - http://bmjopen.bmj.com/site/about/guidelines.xhtml

suggesting that clinical trials and clinical treatment strategies should pay more attention to HRQoL. Besides, a more detailed approach to toxicity penalties that accounts for adverse event is required, as well as an adjusted scoring for those studies with HRQoL.

Funding

None.

Contributors

LD, YW and XY designed the study. XY, ZS and PW extracted the data from all sources, performed the analyses and drafted the manuscript. All authors critically revised the manuscript.

Competing interests

All authors are employees of Betta Pharmaceuticals Co., Ltd and declared no competing interests.

Data availability statement

Data are available upon reasonable request.

Ethics approval

The ethics approval was not needed as the study was an analysis of existing published data.

For peer review only - http://bmjopen.bmj.com/site/about/guidelines.xhtml

References

1. Siegel RL, Miller KD, Fuchs HE, Jemal A. Cancer statistics, 2022. CA Cancer J Clin. 2022;72(1):7-33.

2. Ahmad FB, Anderson RN. The Leading Causes of Death in the US for 2020. JAMA. 2021;325(18):1829-1830.

3. Tarantino P, Carmagnani Pestana R, Corti C, et al. Antibody-drug conjugates: Smart chemotherapy delivery across tumor histologies. CA Cancer J Clin. 2022;72(2):165-182.

4. Chau CH, Steeg PS, Figg WD. Antibody-drug conjugates for cancer. Lancet. 2019;394(10200):793-804.

5. Sievers EL, Larson RA, Stadtmauer EA, et al. Efficacy and safety of gemtuzumab ozogamicin in patients with CD33-positive acute myeloid leukemia in first relapse. J Clin Oncol. 2001;19(13):3244-3254.

6. Fu Z, Li S, Han S, Shi C, Zhang Y. Antibody drug conjugate: the "biological missile" for targeted cancer therapy. Signal Transduct Target Ther. 2022;7(1):93.

7. Schnipper LE, Davidson NE, Wollins DS, et al. American Society of Clinical Oncology Statement: A Conceptual Framework to Assess the Value of Cancer Treatment Options. J Clin Oncol. 2015;33(23):2563-2577.

8. Schnipper LE, Davidson NE, Wollins DS, et al. Updating the American society of clinical oncology value framework: revisions and reflections in response to comments received. J Clin Oncol. 2016;34(24):2925–2933.

9. Cherny NI, Sullivan R, Dafni U, et al. A standardised, generic, validated approach to stratify the magnitude of clinical benefit that can be anticipated from anti-cancer therapies: The European Society for Medical Oncology Magnitude of Clinical Benefit Scale (ESMO-MCBS). Ann Oncol. 2015;26(8):1547–1573.

10. Cherny NI, Dafni U, Bogaerts J, et al. ESMO-Magnitude of Clinical Benefit Scale version 1.1. Ann Oncol. 2017;28(10):2340–2366.

11. Kiesewetter B, Raderer M, Steger GG, et al. The European Society for Medical Oncology Magnitude of Clinical Benefit Scale in daily practice: a single institution,

For peer review only

For peer review only - http://bmjopen.bmj.com/site/about/guidelines.xhtml

real-life experience at the Medical University of Vienna. ESMO Open. 2016;1(4):e000066.

12. Jones D.S., Podolsky S.H. The history and fate of the gold standard. Lancet. 2015;385:1502–1503.

13. Cherny NI, Sullivan R, Dafni U, et al. A standardised, generic, validated approach to stratify the magnitude of clinical benefit that can be anticipated from anti-cancer therapies: the European Society for Medical Oncology Magnitude of Clinical Benefit Scale (ESMO-MCBS). Ann Oncol. 2017;28(11):2901-2905.

14. von Minckwitz G, Huang CS, Mano MS, et al. Trastuzumab Emtansine for Residual Invasive HER2-Positive Breast Cancer. N Engl J Med. 2019;380(7):617-628.

15. Verma S, Miles D, Gianni L, et al. Trastuzumab emtansine for HER2-positive advanced breast cancer. N Engl J Med. 2012;367(19):1783-1791.

16. Diéras V, Miles D, Verma S, et al. Trastuzumab emtansine versus capecitabine plus lapatinib in patients with previously treated HER2-positive advanced breast cancer (EMILIA): a descriptive analysis of final overall survival results from a randomised, open-label, phase 3 trial. Lancet Oncol. 2017;18(6):732-742.

17. Welslau M, Diéras V, Sohn JH, et al. Patient-reported outcomes from EMILIA, a randomized phase 3 study of trastuzumab emtansine (T-DM1) versus capecitabine and lapatinib in human epidermal growth factor receptor 2-positive locally advanced or metastatic breast cancer. Cancer. 2014;120(5):642-651.

18. Powles T, Rosenberg JE, Sonpavde GP, et al. Enfortumab Vedotin in Previously Treated Advanced Urothelial Carcinoma. N Engl J Med. 2021;384(12):1125-1135.

19. Modi S, Saura C, Yamashita T, et al. Trastuzumab Deruxtecan in Previously Treated HER2-Positive Breast Cancer. N Engl J Med. 2020;382(7):610-621.

20. Cortés J, Kim SB, Chung WP, et al. Trastuzumab Deruxtecan versus Trastuzumab Emtansine for Breast Cancer. N Engl J Med. 2022;386(12):1143-1154.

21. Hurvitz SA, Hegg R, Chung WP, et al. Trastuzumab deruxtecan versus trastuzumab emtansine in patients with HER2-positive metastatic breast cancer:

For peer review only

For peer review only - http://bmjopen.bmj.com/site/about/guidelines.xhtml

updated results from DESTINY-Breast03, a randomised, open-label, phase 3 trial. Lancet. 2023;401(10371):105-117.

22. Modi S, Jacot W, Yamashita T, et al. Trastuzumab Deruxtecan in Previously Treated HER2-Low Advanced Breast Cancer. N Engl J Med. 2022;387(1):9-20.

23. Shitara K, Bang YJ, Iwasa S, et al. Trastuzumab Deruxtecan in Previously Treated HER2-Positive Gastric Cancer. N Engl J Med. 2020;382(25):2419-2430.

24. Van Cutsem E, di Bartolomeo M, Smyth E, et al. Trastuzumab deruxtecan in patients in the USA and Europe with HER2-positive advanced gastric or gastroesophageal junction cancer with disease progression on or after a trastuzumab-containing regimen (DESTINY-Gastric02): primary and updated analyses from a single-arm, phase 2 study. Lancet Oncol. 2023;24(7):744-756.

25. Goto K, Goto Y, Kubo T, et al. Trastuzumab Deruxtecan in Patients With HER2-Mutant Metastatic Non-Small-Cell Lung Cancer: Primary Results From the Randomized, Phase II DESTINY-Lung02 Trial. J Clin Oncol. 2023;JCO2301361.

26. Bardia A, Hurvitz SA, Tolaney SM, et al. Sacituzumab Govitecan in Metastatic Triple-Negative Breast Cancer. N Engl J Med. 2021;384(16):1529-1541.

27. Loibl S, Loirat D, Tolaney SM, et al. Health-related quality of life in the phase III ASCENT trial of sacituzumab govitecan versus standard chemotherapy in metastatic triple-negative breast cancer. Eur J Cancer. 2023;178:23-33.

28. Rugo HS, Bardia A, Marmé F, et al. Sacituzumab Govitecan in Hormone Receptor-Positive/Human Epidermal Growth Factor Receptor 2-Negative Metastatic Breast Cancer. J Clin Oncol. 2022;40(29):3365-3376.

29. Rugo HS, Bardia A, Marmé F, et al. Overall survival with sacituzumab govitecan in hormone receptor-positive and human epidermal growth factor receptor 2-negative metastatic breast cancer (TROPiCS-02): a randomised, open-label, multicentre, phase 3 trial. Lancet. 2023;S0140-6736(23)01245-X.

30. Tagawa ST, Balar AV, Petrylak DP, et al. TROPHY-U-01: A Phase II Open-Label Study of Sacituzumab Govitecan in Patients With Metastatic Urothelial Carcinoma Progressing After Platinum-Based Chemotherapy and Checkpoint Inhibitors. J Clin Oncol. 2021;39(22):2474-2485.

For peer review only - http://bmjopen.bmj.com/site/about/guidelines.xhtml

31. Cognetti DM, Johnson JM, Curry JM, et al. Phase 1/2a, open-label, multicenter study of RM-1929 photoimmunotherapy in patients with locoregional, recurrent head and neck squamous cell carcinoma. Head Neck. 2021;43(12):3875-3887.

32. Peng Z, Liu T, Wei J, et al. Efficacy and safety of a novel anti-HER2 therapeutic antibody RC48 in patients with HER2-overexpressing, locally advanced or metastatic gastric or gastroesophageal junction cancer: a single-arm phase II study. Cancer Commun (Lond). 2021;41(11):1173-1182.

33. Sheng X, Yan X, Wang L, et al. Open-label, Multicenter, Phase II Study of RC48-ADC, a HER2-Targeting Antibody-Drug Conjugate, in Patients with Locally Advanced or Metastatic Urothelial Carcinoma. Clin Cancer Res. 2021;27(1):43-51.

34. Coleman RL, Lorusso D, Gennigens C, et al. Efficacy and safety of tisotumab vedotin in previously treated recurrent or metastatic cervical cancer (innovaTV 204/GOG-3023/ENGOT-cx6): a multicentre, open-label, single-arm, phase 2 study. Lancet Oncol. 2021;22(5):609-619.

35. Matulonis UA, Lorusso D, Oaknin A, et al. Efficacy and Safety of Mirvetuximab Soravtansine in Patients With Platinum-Resistant Ovarian Cancer With High Folate Receptor Alpha Expression: Results From the SORAYA Study. J Clin Oncol. 2023;41(13):2436-2445.

36. García-Fumero R, Fernández-López C, Calleja-Hernández MÁ, Expósito-Hernández J. Analyzing the clinical benefit of newer therapies for advanced or metastatic non-small-cell lung cancer: application of the ESMO-magnitude of clinical benefit scale v1.1. Acta Oncol. 2021;60(9):1225-1232.

37. George S.L. Response rate as an endpoint in clinical trial. J Natl Cancer Inst. 2007;99:98–99.

38. Chen EY, Joshi SK, Tran A, Prasad V. Estimation of Study Time Reduction Using Surrogate End Points Rather Than Overall Survival in Oncology Clinical Trials. JAMA Intern Med. 2019;179(5):642-647.

39. Gyawali B, Hey SP, Kesselheim AS. Assessment of the clinical benefit of cancer drugs receiving accelerated approval. JAMA Intern Med. 2019;179(7):906-913.

For peer review only - http://bmjopen.bmj.com/site/about/guidelines.xhtml

40. Pignatti F, Wilking U, Postmus D, et al. The value of anticancer drugs-a regulatory view. Nat Rev Clin Oncol. 2022;19:207–215.

41. Tibau A, Molto C, Borrell M, et al. Magnitude of Clinical Benefit of Cancer Drugs Approved by the US Food and Drug Administration Based on Single-Arm Trials. JAMA Oncol. 2018;4(11):1610-1611.

42. Gyawali B, de Vries EGE, Dafni U, et al. Biases in study design, implementation, and data analysis that distort the appraisal of clinical benefit and ESMO-Magnitude of Clinical Benefit Scale (ESMO-MCBS) scoring. ESMO Open. 2021;6(3):100117.

43. Davis C, Naci H, Gurpinar E, Poplavska E, Pinto A, Aggarwal A. Availability of evidence of benefits on overall survival and quality of life of cancer drugs approved by European Medicines Agency: retrospective cohort study of drug approvals 2009-13. BMJ. 2017;359:j4530.

44. Naci H, Davis C, Savović J, et al. Design characteristics, risk of bias, and reporting of randomised controlled trials supporting approvals of cancer drugs by European Medicines Agency, 2014-16: cross sectional analysis. BMJ. 2019;366:l5221.

45. Hilal T, Gonzalez-Velez M, Prasad V. Limitations in Clinical Trials Leading to Anticancer Drug Approvals by the US Food and Drug Administration. JAMA Intern Med. 2020;180(8):1108-1115.

46. Kiesewetter B, Dafni U, de Vries EGE, et al. ESMO-Magnitude of Clinical Benefit Scale for haematological malignancies (ESMO-MCBS:H) version 1.0. Ann Oncol. 2023;34(9):734-771.

For peer review only

For peer review only - http://bmjopen.bmj.com/site/about/guidelines.xhtml

Table 1 Characteristics of the included studies

Drug name	Trial name/ Registry number	Design	Tumour type	First approval data	First approval agency	Ref
Trastuzumab emtansine (T-DM1)	KATHERINE NCT01772472	Phase 3 RCT	HER2-positive early breast cancer who had residual invasive disease in the breast or axilla at surgery after receiving neoadjuvant therapy containing a taxane (with or without anthracycline) and trastuzumab	2013	FDA	14
	EMILIA NCT00829166	Phase 3 RCT	HER2-positive advanced breast cancer second line	2019	FDA	15-17
Enfortumab vedotin	EV-301 NCT03474107	Phase 3 RCT	Locally advanced or metastatic urothelial carcinoma	2019	FDA	18
Trastuzumab deruxtecan (DS-8201)	DESTINY-Breast01 NCT03248492	Phase 2 SAT	HER2-positive metastatic breast cancer third line	2021	FDA	19
	DESTINY-Breast03 NCT03529110	Phase 3 RCT	HER2-positive metastatic breast cancer second line	2022	FDA	20, 21
	DESTINY-Breast04 NCT03734029	Phase 3 RCT	HER2-low metastatic breast cancer	2022	FDA	22
	DESTINY-Gastric01 NCT03329690	Phase 2 RCT	HER2-positive advanced gastric cancer	2021	FDA	23
	DESTINY-Gastric02 NCT04014075	Phase 2 SAT	HER2-positive advanced gastric or gastroesophageal junctionadenocarcinoma after a prior trastuzumab-based regimen	2023	EMA	24
	DESTINY-Lung02 NCT04644237	Phase 2 SAT	Metastatic HER2-mutant NSCLC second line	2022	FDA	25
Sacituzumab govitecan	ASCENT NCT02574455	Phase 3 RCT	Relapsed or refractory metastatic triple-negative breast cancer	2020	FDA	26, 27
	TROPiCS-02 NCT03901339	Phase 3 RCT	Endocrine-resistant, chemotherapy- treated HR+/HER2- locally recurrent inoperable or metastatic breast cancer	2023	FDA	28, 29
	TROPHY-U-01 NCT03547973	Phase 2 RCT	Metastatic urothelial carcinoma	2021	FDA	30
Cetuximab saratolacan	- NCT02422979	Phase 2 SAT	Recurrent head and neck squamous cell carcinoma	2020	PMDA	31

For peer review only

Disitamab vedotin	- NCT03556345	Phase 2 SAT	HER2-overexpressing, locally advanced or metastatic gastric or gastroesophageal junction cancer	2021	NMPA	32
	- NCT03507166	Phase 2 SAT	HER2+ locally advanced or metastatic urothelial carcinoma	2022	NMPA	33
Tisotumab vedotin	innovaTV 204 NCT03438396	Phase 2 SAT	Recurrent or metastatic cervical cancer	2021	FDA	34
Mirvetuximab soravtansine	SORAYA NCT04296890	Phase 2 SAT	FRa-high, platinum-resistant epithelial ovarian cancer	2022	FDA	35

RCT, randomized control trial; SAT, single-arm trial

For peer review only

Table 2 Clinical benefit according to ESMO-MCBS v1.1 and concordance rate between ESMO Framework in this study and ESMO-MCBS scorecards

Trial name	Intervention group	PO	PO ctrl group	PO gain	PO HR (95% CI)	Toxicity/ QoL	ESM0-MCBS v1.1	Scorecards
KATHERINE	T-DM1	iDFS	3-year: 77.0%	3-year: 11.3%	0.50 (0.39-0.64)	NA	A (form 1)	A
EMILIA	T-DM1	PFS and OS	25.9 mo	4.0 mo	0.75 (0.64-0.88)	Improved HRQoL	3 (form 2a)	4
EV-301	Enfortumab vedotin	OS	8.97 mo	3.91 mo	0.70 (0.56-0.89)	NA	4 (form 2a)	4
DESTINY-Breast01*	DS-8201	ORR	-	-	-	NA	3 (form 3)	2
DESTINY-Breast03	DS-8201	PFS (OS improved)	2-year: 69.9%	2-year: 7.5%	0.64 (0.47-0.87)	NA	3 (form 2a)	4
DESTINY-Breast04	DS-8201	PFS (OS improved)	16.8 mo	6.6 mo	0.64 (0.49–0.84)	NA	4 (form 2a)	4
DESTINY-Gastric01#	DS-8201	ORR	14%	37%	-	NA	2 (form 2c)	4
DESTINY-Gastric02#	DS-8201	ORR	-	-	-	NA	2 (form 3)	2
DESTINY-Lung02*	DS-8201	ORR	-	-	-	NA	3 (form 3)	NA
ASCENT	Sacituzumab govitecan	PFS (OS improved)	6.9 mo	4.9 mo	0.51 (0.41-0.62)	Increased toxicity/ Improved HRQoL	4 (form 2a)	4
TROPiCS-02	Sacituzumab govitecan	PFS (OS improved)	11.2 mo	3.2 mo	0.79 (0.65-0.96)	NA	1 (form 2a)	3
TROPHY-U-01*	Sacituzumab govitecan	ORR	-	-	-	NA	3 (form 3)	NA
NCT02422979*	Cetuximab saratolacan	ORR	-	-	-	NA	2 (form 3)	NA
NCT03556345*	Disitamab vedotin	ORR	-	-	-	NA	2 (form 3)	NA
NCT03507166*	Disitamab vedotin	ORR	-	-	-	NA	3 (form 3)	NA

For peer review only

innovaTV 204[*]	Tisotumab vedotin	ORR	-	-	-	NA	2 (form 3)	2
SORAYA[*]	Mirvetuximab soravtansine	ORR	-	-	-	NA	2 (form 3)	2

PO, primary outcome;

For peer review only

For peer review only - http://bmjopen.bmj.com/site/about/guidelines.xhtml

Supplemental Table S1 Search strategy for studies on ADCs in solid tumours

Database	Search Period	Search Terms
Pubmed	2000 to 18 October 2023	1. antibody-drug conjugates<br>2. solid cancer<br>3. approve<br>4. clinical trials<br>5. quality of life<br>6. follow-up survival<br>7. updated overall survival

For peer review only

PRISMA 2020 Checklist

Section and Topic	Item #	Checklist item	Location where item is reported
**TITLE**			
Title	1	Identify the report as a systematic review.	Page 1
**ABSTRACT**			
Abstract	2	See the PRISMA 2020 for Abstracts checklist.	Page 2
**INTRODUCTION**			
Rationale	3	Describe the rationale for the review in the context of existing knowledge.	Page 4
Objectives	4	Provide an explicit statement of the objective(s) or question(s) the review addresses.	Page 4
**METHODS**			
Eligibility criteria	5	Specify the inclusion and exclusion criteria for the review and how studies were grouped for the syntheses.	Page 5
Information sources	6	Specify all databases, registers, websites, organisations, reference lists and other sources searched or consulted to identify studies. Specify the date when each source was last searched or consulted.	Page 5
Search strategy	7	Present the full search strategies for all databases, registers and websites, including any filters and limits used.	Page 5 Supplemental Table S1
Selection process	8	Specify the methods used to decide whether a study met the inclusion criteria of the review, including how many reviewers screened each record and each report retrieved, whether they worked independently, and if applicable, details of automation tools used in the process.	Page 5
Data collection process	9	Specify the methods used to collect data from reports, including how many reviewers collected data from each report, whether they worked independently, any processes for obtaining or confirming data from study investigators, and if applicable, details of automation tools used in the process.	Page 5
Data items	10a	List and define all outcomes for which data were sought. Specify whether all results that were compatible with each outcome domain in each study were sought (e.g. for all measures, time points, analyses), and if not, the methods used to decide which results to collect.	Page 5
	10b	List and define all other variables for which data were sought (e.g. participant and intervention characteristics, funding sources). Describe any assumptions made about any missing or unclear information.	Page 5
Study risk of bias assessment	11	Specify the methods used to assess risk of bias in the included studies, including details of the tool(s) used, how many reviewers assessed each study and whether they worked independently, and if applicable, details of automation tools used in the process.	Page 5
Effect measures	12	Specify for each outcome the effect measure(s) (e.g. risk ratio, mean difference) used in the synthesis or presentation of results.	Page 5-6
Synthesis methods	13a	Describe the processes used to decide which studies were eligible for each synthesis (e.g. tabulating the study intervention characteristics and comparing against the planned groups for each synthesis (item #5)).	NA
	13b	Describe any methods required to prepare the data for presentation or synthesis, such as handling of missing summary statistics, or data conversions.	NA
	13c	Describe any methods used to tabulate or visually display results of individual studies and syntheses.	NA
	13d	Describe any methods used to synthesize results and provide a rationale for the choice(s). If meta-analysis was performed, describe the model(s), method(s) to identify the presence and extent of statistical heterogeneity, and software package(s) used.	NA
	13e	Describe any methods used to explore possible causes of heterogeneity among study results (e.g. subgroup analysis, meta-regression).	NA
	13f	Describe any sensitivity analyses conducted to assess robustness of the synthesized results.	NA
Reporting bias	14	Describe any methods used to assess risk of bias due to missing results in a synthesis (arising from reporting biases).	NA

For peer review only - http://bmjopen.bmj.com/site/about/guidelines.xhtml

PRISMA 2020 Checklist

Section and Topic	Item #	Checklist item	Location where item is reported
assessment			
Certainty assessment	15	Describe any methods used to assess certainty (or confidence) in the body of evidence for an outcome.	NA
**RESULTS**			
Study selection	16a	Describe the results of the search and selection process, from the number of records identified in the search to the number of studies included in the review, ideally using a flow diagram.	Page 6
	16b	Cite studies that might appear to meet the inclusion criteria, but which were excluded, and explain why they were excluded.	Page 6
Study characteristics	17	Cite each included study and present its characteristics.	Page 6, Table 1
Risk of bias in studies	18	Present assessments of risk of bias for each included study.	NA
Results of individual studies	19	For all outcomes, present, for each study: (a) summary statistics for each group (where appropriate) and (b) an effect estimate and its precision (e.g. confidence/credible interval), ideally using structured tables or plots.	Page 6, Table 1
Results of syntheses	20a	For each synthesis, briefly summarise the characteristics and risk of bias among contributing studies.	NA
	20b	Present results of all statistical syntheses conducted. If meta-analysis was done, present for each the summary estimate and its precision (e.g. confidence/credible interval) and measures of statistical heterogeneity. If comparing groups, describe the direction of the effect.	NA
	20c	Present results of all investigations of possible causes of heterogeneity among study results.	NA
	20d	Present results of all sensitivity analyses conducted to assess the robustness of the synthesized results.	NA
Reporting biases	21	Present assessments of risk of bias due to missing results (arising from reporting biases) for each synthesis assessed.	NA
Certainty of evidence	22	Present assessments of certainty (or confidence) in the body of evidence for each outcome assessed.	NA
**DISCUSSION**			
Discussion	23a	Provide a general interpretation of the results in the context of other evidence.	Page 7
	23b	Discuss any limitations of the evidence included in the review.	Page 10
	23c	Discuss any limitations of the review processes used.	Page 10
	23d	Discuss implications of the results for practice, policy, and future research.	Page 9-10
**OTHER INFORMATION**			
Registration and protocol	24a	Provide registration information for the review, including register name and registration number, or state that the review was not registered.	Page 5
	24b	Indicate where the review protocol can be accessed, or state that a protocol was not prepared.	Page 5
	24c	Describe and explain any amendments to information provided at registration or in the protocol.	NA
Support	25	Describe sources of financial or non-financial support for the review, and the role of the funders or sponsors in the review.	Page 11
Competing interests	26	Declare any competing interests of review authors.	Page 11
Availability of data, code and	27	Report which of the following are publicly available and where they can be found: template data collection forms; data extracted from included studies; data used for all analyses; analytic code; any other materials used in the review.	Page 11

For peer review only

PRISMA 2020 Checklist

Section and Topic	Item #	Checklist item	Location where item is reported
other materials			

From: Page MJ, McKenzie JE, Bossuyt PM, Boutron I, Hoffmann TC, Mulrow CD, et al. The PRISMA 2020 statement: an updated guideline for reporting systematic reviews. BMJ 2021;372:n71. doi: 10.1136/bmj.n71

For more information, visit: http://www.prisma-statement.org/

For peer review only

BMJ Open

Application of the ESMO Magnitude of Clinical Benefit Scale to assess the clinical benefit of antibody–drug conjugates in solid cancer: a systematic descriptive analysis of phase 3 and pivotal phase 2 trials

Journal:	*BMJ Open*
Manuscript ID	bmjopen-2023-077108.R2
Article Type:	Original research
Date Submitted by the Author:	01-Feb-2024
Complete List of Authors:	Ding, Lieming; Betta Pharmaceuticals Co Ltd<br>Yuan, Xiaobin; Betta Pharmaceuticals Co Ltd<br>Wang, Yang; Betta Pharmaceuticals Co Ltd<br>Shen, Zhilin; Betta Pharmaceuticals Co Ltd,<br>Wu, Pengxiang; Betta Pharmaceuticals Co Ltd
**Primary Subject Heading**:	Oncology
Secondary Subject Heading:	Global health
Keywords:	ONCOLOGY, PUBLIC HEALTH, Patient-Centered Care

SCHOLARONE™
Manuscripts

I, the Submitting Author has the right to grant and does grant on behalf of all authors of the Work (as defined in the below author licence), an exclusive licence and/or a non-exclusive licence for contributions from authors who are: i) UK Crown employees; ii) where BMJ has agreed a CC-BY licence shall apply, and/or iii) in accordance with the terms applicable for US Federal Government officers or employees acting as part of their official duties; on a worldwide, perpetual, irrevocable, royalty-free basis to BMJ Publishing Group Ltd ("BMJ") its licensees and where the relevant Journal is co-owned by BMJ to the co-owners of the Journal, to publish the Work in this journal and any other BMJ products and to exploit all rights, as set out in our licence.

The Submitting Author accepts and understands that any supply made under these terms is made by BMJ to the Submitting Author unless you are acting as an employee on behalf of your employer or a postgraduate student of an affiliated institution which is paying any applicable article publishing charge ("APC") for Open Access articles. Where the Submitting Author wishes to make the Work available on an Open Access basis (and intends to pay the relevant APC), the terms of reuse of such Open Access shall be governed by a Creative Commons licence – details of these licences and which Creative Commons licence will apply to this Work are set out in our licence referred to above.

Other than as permitted in any relevant BMJ Author's Self Archiving Policies, I confirm this Work has not been accepted for publication elsewhere, is not being considered for publication elsewhere and does not duplicate material already published. I confirm all authors consent to publication of this Work and authorise the granting of this licence.

For peer review only

Application of the ESMO Magnitude of Clinical Benefit Scale to assess the clinical benefit of antibody–drug conjugates in solid cancer: a systematic descriptive analysis of phase 3 and pivotal phase 2 trials

Lieming Ding[1,* †], Xiaobin Yuan[1, *], Yang Wang[1], Zhilin Shen[1], Pengxiang Wu[1]

[1]Betta Pharmaceuticals Co., Ltd, Hangzhou, China

[*]These authors contributed equally

[†]Correspondence to:

Dr. Lieming Ding

E-mail: lieming.ding@bettapharma.com

For peer review only - http://bmjopen.bmj.com/site/about/guidelines.xhtml

Abstract

Objective: The aim of this study was to assess the clinical benefit value of approved antibody-drug conjugates (ADCs) for solid tumours using the European Society for Medical Oncology Magnitude of Clinical Benefit Scale (ESMO-MCBS) v1.1.

Design: Systematic descriptive analysis.

Data sources: PubMed was searched for publications from January 1 2000 to 18 October 2023.

Eligibility criteria: We included the phase 3 randomised controlled trials or phase 2 pivotal trials leading to approval of ADCs in solid tumours.

Data extraction and synthesis: Two independent reviewers extracted data and discrepancies were resolved by consensus in the presence of a third investigator.

Results: ESMO-MCBS scores were calculated for 16 positive clinical trials of eight ADCs, which were firstly approved by US Food and Drug Administration (FDA), European Medicines Agency (EMA), China National Medical Products Administration (NMPA) and Japanese Pharmaceuticals and Medical Devices Agency (PMDA) for solid cancers. Among 16 trials, four (25%) met the ESMO-MCBS benefit threshold grade, while 12 (75%) of the regimens did not meet the ESMO-MCBS benefit threshold grade. Five (31%) of the 16 trials had no published scorecard on the ESMO website due to the approval by other jurisdictions but not by the FDA or EMA. Discrepancies between our results and the ESMO scorecard were observed in four (36%) of 11 trials, mostly owing to integration of more recent data.

Conclusions: ESMO-MCBS is an important tool for assessing clinical benefit of cancer drugs, but not all drugs met the meaningful benefit threshold.

For peer review only - http://bmjopen.bmj.com/site/about/guidelines.xhtml

STRENGTHS AND LIMITATIONS OF THIS STUDY

- This analysis assessed clinical benefit in phase 3 randomised controlled trials or phase 2 pivotal trials leading to approval.
- ESMO-MCBS is a validated tool for grading the clinical benefit of novel drugs.
- Shortcomings of ESMO-MCBS were identified, which should be addressed in future versions.
- The data lack some important information such as the drug costs analysis and ASCO Value Framework.

For peer review only

For peer review only - http://bmjopen.bmj.com/site/about/guidelines.xhtml

INTRODUCTION

Cancer is one of the leading causes of the death worldwide and ranks second in mortality below heart disease in the US [1, 2]. Considering the off-target toxicities of chemotherapeutic drugs, novel anti-cancer agents which can selectively target cancer cells and attenuate side effects are urgent. Antibody-drug conjugates (ADCs) are the emerging next generation therapeutics after monoclonal antibodies (mAbs), attributing to the superior anti-cancer activity over traditional chemotherapy [3, 4]. In 2000, US Food and Drug Administration (FDA) approved the first ADC, gemtuzumab ozogamicin (Mylotarg), for the treatment of patients with acute myeloid leukemia (AML) [5]. To date, a total of 15 ADCs have been approved worldwide and more than 100 ADCs are currently being evaluated in clinical trials [6].

The paradigm of cancer therapy has shifted from the disease-centred strategy to a patient-centred strategy, emphasizing the comprehensive value of the therapeutic regimen, including quality of life (QoL) and cost. However, it is always hard to objectively evaluate therapy value and clinical benefit. Both the American Society of Clinical Oncology Value Framework (ASCO-VF) [7, 8] and the European Society for Medical Oncology Magnitude of Clinical Benefit Scale (ESMO-MCBS) [9, 10] aimed to evaluate the clinical benefit of novel anti-cancer treatments. Besides, the ESMO-MCBS seems to be very reliable in advanced or metastatic diseases across all therapeutic settings in daily practice [11].

Randomised controlled trials are regarded as the 'gold standard' in evaluating medicinal products [12]. Considering an unmet medical need, single-arm trials can also support approvals, although uncommon. Here, we overview the landscape of ADCs approved by FDA, European Medicines Agency (EMA), China National Medical Products Administration (NMPA) and Japanese Pharmaceuticals and Medical Devices Agency (PMDA) for solid cancer, and describe the clinical benefit of these ADCs and further explore whether clinical benefit is related to the high-quality cancer care.

METHODS

For peer review only - http://bmjopen.bmj.com/site/about/guidelines.xhtml

Design

Our study is a systematic descriptive analysis. There was no protocol and the study was not registered.

Data sources and searches

We identified all approved indications of eight ADCs between January 1, 2000 and 18 October, 2023 by searching different official websites, including FDA (https://www.fda.gov/), EMA (http://www.ema.europa.eu), NMPA (https://www.nmpa.gov.cn/) and PMDA (https://www.pmda.go.jp/english/index.html). Drug names, indications and approval dates were recorded from these official websites. In addition, PubMed (https://pubmed.ncbi.nlm.nih.gov/) was searched for publications from January 1 2000 to 18 October, 2023. The full search terms used in this study are presented in supplementary table S1. The search was limited to human studies and had no language restrictions.

Study selection

The inclusion criterions were the phase 2 and phase 3 randomised controlled trials that led to approval by regulatory authorities. The phase 2 single-arm trials were included if they were pivotal to obtain its marketing approval. Updated data of pivotal studies or reports on toxicity or QoL outcomes were also considered.

The exclusion criteria were: non-pivotal trials (e.g. the secondary, subset, meta-analyses or systematic reviews; phase 1 or 4 trials; exploratory trials); prematurely stopped RCTs; no drug therapy intervention; haematological cancers; non-English articles.

Data extraction

The study was reviewed independently by two investigators (XY and YW) and extracted relevant details from each study, including study design, sample size and endpoints (objective response rate [ORR], disease-free survival [DFS], progression free survival [PFS], overall survival [OS], QoL and toxicity). The discrepancies were resolved by consensus in the presence of a third investigator (LD).

ESMO-MCBS scoring

The ESMO-MCBS can be used to rank the value of systemic therapies based on

For peer review only - http://bmjopen.bmj.com/site/about/guidelines.xhtml

reported relative and absolute benefits in terms of improved survival (DFS, PFS and OS) and better survival (e.g. QoL, toxicity). It is worth noting that the ESMO-MCBS can only be applied to studies showing statistically significant differences. For the non-curative setting, form 2a and form 2b are available, considering the the predefined primary and secondary endpoints with regard to absolute gain as well as the lower end of the 95% confidence interval (CI) of the corresponding hazard ratio (HR). In the context of non-inferiority trials, form 2c has been devised, incorporating QoL and toxicity data to evaluate scores. Additionally, form 3 is designated to assess single-arm studies. Initial score was adjusted based on various ESMO criteria, including long-term survival, toxicity and QoL [13]. Palliative therapies were ultimately graded 1-5 for advanced disease setting and as A, B, or C for adjuvant or neoadjuvant therapy setting, with scores 5, 4, A or B representing substantial clinical benefit [14].

Patient and public involvement

None.

RESULTS

Trial selection and characteristics

After excluding 7 of 15 approved ADCs (i.e. gemtuzumab ozogamicin, brentuximab vedotin, inotuzumab ozogamicin, moxetumomab pasudotox, polatuzumab vedotin, belantamab mafodotin and loncastuximab tesirine) for haematological cancers, clinical trials on 8 ADCs for solid cancers were identified. A total of 21 publications from 16 clinical trials (9 randomized controlled trials and 7 single-arm trials) leading to approval for solid cancers were finally identified, including 2 for trastuzumab emtansine, 1 for enfortumab vedotin, 5 for trastuzumab deruxtecan, 3 for sacituzumab govitecan, 1 for cetuximab saratolacan, 2 for disitamab vedotin, 1 for tisotumab vedotin and 1 for mirvetuximab soravtansine. The approved ADCs covered 7 solid cancer types, including breast cancer, urothelial carcinoma, gastric cancer, non-small cell lung cancer, head-and-neck squamous cell carcinoma, cervical cancer and ovarian cancer (table 1). The publications on updated efficacy or QoL data were also included

For peer review only - http://bmjopen.bmj.com/site/about/guidelines.xhtml

for ESMO-MCBS score adjustment [15-35].

ESMO-MCBS scores

In total, 16 clinical trials were assessed by form 1 (n = 1), form 2a (n = 6), form 2c (n = 1) and form 3 (n = 8). Among 16 trials, 4 (25%) of the regimens met the ESMO-MCBS benefit threshold grade, while 12 (75%) of the regimens did not meet the ESMO-MCBS benefit threshold grade (table 2).

In trials meeting the ESMO threshold for clinically meaningful benefit (scores 4-5), the median PFS and median OS seemed slightly extended in contrast to those falling below the threshold (scores 1-3), despite that statistically significant differences were not calculated.

Upon the comparison of our final ESMO-MCBS scores with the relevant scorecards accessible through the ESMO website, 5 out of 16 (31%) trials did not yet have the published scorecard, mostly because they are approved by jurisdictions other than the EMA and FDA. In our study, several ADCs have been only approved by local agencies in China and Japan. Discrepancies were observed in 4 of 11 (36%) trials (table 2). We find different scores mostly due to integration of more recent data. When comes to multiple publications, the clinical benefit was evaluated based on both the primary and secondary manuscripts. While we were always presented the scores according to the newly published articles due to the more mature OS data or QoL data. Interestingly, discrepancies were observed in EMILIA and TROPiCS trials. The scores in EMILIA based on the interim analyses and final OS analyses were 4 and 3, respectively. The score was decreased as the OS HR was increased. Similarly, the scores in TROPiCS-02 based on the interim analyses and final OS analyses were 3 and 1, respectively. As OS data was mature and showed benefit, the form 2b was replaced by form 2a; however, the OS HR was increased, leading the low score. Besides, in the DESTINY-Breast03 study, given that the form 2b was replaced by form 2a, we ignored the long-term plateau in the PFS curve, resulting in the discrepancy. According to the ESMO-MCBS scorecard, DESTINY-Gastric01 was evaluated as 4 with the form 2a instead of form 2c, and the main reason was that the hierarchical primary outcome was OS.

For peer review only - http://bmjopen.bmj.com/site/about/guidelines.xhtml

DISCUSSION

To the best of our knowledge, this is the first review to apply ESMO-MCBS v1.1 exclusively to approved ADCs. In this study, we utilized the ESMO-MCBS tool to gauge the extent of clinical benefit derived from phases 2 and 3 trials on ADC therapy for solid tumours are translated. Our cohort of 16 clinical trials encompassed multiple therapeutic options with ADCs from 2000 to 2023.

Despite the preferred trials for the application of marketing approval, randomized controlled trials are not always likely to perform. In recent years, various drugs have been approved based on the single-arm trials. These approvals were instituted to fulfil an unmet medical necessity, with less comprehensive data compared to those for standard approval processes. However, the evidence level that the investigational drugs provide clinical benefits may be insufficiency when solely based on single-arm trials. In our study, to evaluate whether the authorized drugs based on single-arm trials provided substantial benefit, we included these pivotal phase 2 single-arm trials leading to the approval.

The application of ESMO-MCBS forms on the basis of OS and/or PFS was less than those based on ORR (38% vs. 56%). In 5 trials with the primary endpoint of PFS, 4 (80%) trials were assessed using form 2a instead of form 2b due to the OS advantage. However, a previous study revealed that conclusions based on PFS outcomes in these clinical trials might be overestimated by this scoring system, due to the statistical power to only detect significant differences in PFS, rather than OS [14]. ORR is not a direct measure of clinical benefit; however, it is a measure of anti-cancer activity [36]. A study found that 38% and 34% of anti-cancer drugs were approved by FDA based on ORR and PFS, shortening the development durations by 19 months and 11 months, respectively [37]. Nevertheless, among 93 anti-cancer drugs with accelerated approvals, only 20% demonstrated an improvement in OS in confirmatory trials [38]. As previously revealed, the definition of clinical value is different between stakeholders, causing different conclusions [39]. For instance, the ESMO considers benefit as ‘living longer and/or living better’, which resonates in the ESMO-MCBS

For peer review only - http://bmjopen.bmj.com/site/about/guidelines.xhtml

form for single-arm trials [9, 10]. This is confirmed in our findings that the benefit of the majority of ADCs was 'modest' according to ESMO-MCBS scores. A high ESMO-MCBS score for single-arm trials was related to favorable efficacy in combination with an improved QoL [40]. Yet, delayed publications or publication bias for QoL is overlooked by ESMO-MCBS [41]. Therefore, QoL appears of lesser importance in regulatory decision making on single-arm trials.

Despite a minor number of inconsistencies between our final scores and the relevant ESMO scorecards, the final scores varied significantly due to these disparities. The re-assessment of the ESMO-MCBS score is important when results from confirmatory trials are published, which may affect ESMO-MCBS score. Furthermore, extended follow-up may also affect the EMSO-MCBS score. For example, we previously assigned an ESMO-MCBS score of '3' for the sacituzumab govitecan (ASCENT trial) due to the increased toxicity; however, we finally assigned a score of '4' due to the a recent publication on health-related QoL [27]. On the contrary, the score was found to decrease as OS data was mature and showed benefit while the value of OS HR was increased in EMILIA [16, 17] and TROPiCS trials [28, 29]. ESMO-MCBS can develop specialized form for re-evaluation of the updated OS data. Additionally, adjustments sometimes might be confusing. For example, we assigned an ESMO-MCBS score of '3' for the trastuzumab deruxtecan (DESTINY-Breast03 trial) based on the form 2a due to the improved OS; however, ESMO-MCBS scorecard showed '4' due to the long-term plateau in the PFS curve based on form 2b. Although grades ≥3 adverse events may be different between treatment arms, it was not always statistically significant in publications. Besides, adverse events that can affect daily well-being of patients may not be consistently published. Clearer definitions or quantifications of toxicity profiles in future ESMO-MCBS versions are highly desirable. Furthermore, several trials had no published scorecard, because they are approved by jurisdictions other than the EMA and FDA. We suggest that ESMO-MCBS scorecards can also published the drugs that are approved by other agencies other than FDA and EMA.

Almost half of positive clinical trials are unable to demonstrate a substantial clinical

For peer review only - http://bmjopen.bmj.com/site/about/guidelines.xhtml

benefit based on the ESMO framework, and this has drawn criticism in light of market approvals for anti-cancer drugs in recent periods [42-44]. In our study, only 4/16 (25%) of the clinical trials can meet the ESMO-MCBS threshold for clinical benefit. Notably, all single-arm trial cannot meet the ESMO-MCBS threshold for clinical benefit. Form 3 is used to assess single-arm trials, but it should also be noted that by utilizing this form, only grades from 1 to 4 can be attained. As a result, the threshold can only be met if the study demonstrates HRQoL improvements, or additionally, if confirmatory phase 4 trials are accessible.

Furthermore, in a head-to-head trial (DESTINY-Breast03) to compare the efficacy and safety of trastuzumab deruxtecan with those of trastuzumab emtansine in patients with HER2-positive metastatic breast cancer, trastuzumab deruxtecan showed a superior OS over trastuzumab emtansine [21, 22]. Thus, we also confused that whether the OS gain in ESMO-MCBS tool can evaluate the benefits of different drugs in the same indication or the same drug in different indications. The thresholds for the OS gain appear a bit lenient.

This study has several implications. Firstly, this study showed clinical benefits for limited ADCs in solid tumours, suggesting that subsequent clinical trials on the treatment of solid tumours with ADCs should follow the cases with meaningful clinical benefit. Secondly, value frameworks can help not only identify drugs with low or uncertain clinical benefit that should be targeted for price negotiations, but also therapies with evidence of higher clinical benefit to improve access to benefit drugs, thereby contributing to patient-centred cancer treatment goals. Finally, the ADCs with HRQoL improvement showed clinical benefit, suggesting that HRQoL should be paid sufficient attention in clinical trials and clinical treatment strategies.

There are several limitations in our study. First, we limited our analysis to solid tumours and excluded drugs approved to treat hematologic malignancies. When this study was completed in June 2023, the haematology-specific version has yet to be published. We will further access the clinical benefits of ADCs for hematologic malignancies according to the currently published ESMO-MCBS:H v 1.0 [45]. Another main limitation is publication selection bias. However, we implemented a

For peer review only - http://bmjopen.bmj.com/site/about/guidelines.xhtml

For peer review only

rigorous search strategy to mitigate it. Publications were selected by two investigators independently based on predefined inclusion and exclusion criteria. We specifically excluded phase 1 and non-pivotal phase 2 trials to evaluate novel therapies with sufficient efficacy and toxicity data, thereby primarily including authorised regimens applied in routine clinical practice. Furthermore, the limited toxicity data accessible within published clinical trials hindered our ability to accurately adjust preliminary grades. Additionally, our study was also limited by the lack of drug costs, which may help clinicians to select the optimal therapeutic drugs developed for the same clinical entity [23].

In conclusion, this is the first study that used ESMO-MCBS tool to assess clinical benefit of ADCs across several solid cancers. ESMO-MCBS are important tools for assessing clinical benefit of cancer drugs, but not all drugs met the meaningful benefit threshold. Those therapeutic regimens with improved HRQoL showed clinical value, suggesting that clinical trials and clinical treatment strategies should pay more attention to HRQoL. Furthermore, a more exhaustive rule to toxicity penalties and adjusted scoring for HRQoL is warranted.

Funding

None.

Contributors

LD, YW and XY designed the study. XY, ZS and PW extracted the data from all sources, performed the analyses and drafted the manuscript. All authors critically revised the manuscript.

Competing interests

All authors are employees of Betta Pharmaceuticals Co., Ltd.

Data availability statement

Data are available upon reasonable request.

For peer review only - http://bmjopen.bmj.com/site/about/guidelines.xhtml

Ethics approval

Ethics approval was not needed as the study was an analysis of existing published data.

For peer review only - http://bmjopen.bmj.com/site/about/guidelines.xhtml

References

1. Siegel RL, Miller KD, Fuchs HE, Jemal A. Cancer statistics, 2022. CA Cancer J Clin. 2022;72(1):7-33.

2. Ahmad FB, Anderson RN. The Leading Causes of Death in the US for 2020. JAMA. 2021;325(18):1829-1830.

3. Tarantino P, Carmagnani Pestana R, Corti C, et al. Antibody-drug conjugates: Smart chemotherapy delivery across tumor histologies. CA Cancer J Clin. 2022;72(2):165-182.

4. Chau CH, Steeg PS, Figg WD. Antibody-drug conjugates for cancer. Lancet. 2019;394(10200):793-804.

5. Sievers EL, Larson RA, Stadtmauer EA, et al. Efficacy and safety of gemtuzumab ozogamicin in patients with CD33-positive acute myeloid leukemia in first relapse. J Clin Oncol. 2001;19(13):3244-3254.

6. Fu Z, Li S, Han S, Shi C, Zhang Y. Antibody drug conjugate: the "biological missile" for targeted cancer therapy. Signal Transduct Target Ther. 2022;7(1):93.

7. Schnipper LE, Davidson NE, Wollins DS, et al. American Society of Clinical Oncology Statement: A Conceptual Framework to Assess the Value of Cancer Treatment Options. J Clin Oncol. 2015;33(23):2563-2577.

8. Schnipper LE, Davidson NE, Wollins DS, et al. Updating the American society of clinical oncology value framework: revisions and reflections in response to comments received. J Clin Oncol. 2016;34(24):2925–2933.

9. Cherny NI, Sullivan R, Dafni U, et al. A standardised, generic, validated approach to stratify the magnitude of clinical benefit that can be anticipated from anti-cancer therapies: The European Society for Medical Oncology Magnitude of Clinical Benefit Scale (ESMO-MCBS). Ann Oncol. 2015;26(8):1547–1573.

10. Cherny NI, Dafni U, Bogaerts J, et al. ESMO-Magnitude of Clinical Benefit Scale version 1.1. Ann Oncol. 2017;28(10):2340–2366.

11. Kiesewetter B, Raderer M, Steger GG, et al. The European Society for Medical Oncology Magnitude of Clinical Benefit Scale in daily practice: a single institution,

For peer review only - http://bmjopen.bmj.com/site/about/guidelines.xhtml

real-life experience at the Medical University of Vienna. ESMO Open. 2016;1(4):e000066.

12. Jones D.S., Podolsky S.H. The history and fate of the gold standard. Lancet. 2015;385:1502–1503.

13. Cherny NI, Sullivan R, Dafni U, et al. A standardised, generic, validated approach to stratify the magnitude of clinical benefit that can be anticipated from anti-cancer therapies: the European Society for Medical Oncology Magnitude of Clinical Benefit Scale (ESMO-MCBS). Ann Oncol. 2017;28(11):2901-2905.

14. García-Fumero R, Fernández-López C, Calleja-Hernández MÁ, Expósito-Hernández J. Analyzing the clinical benefit of newer therapies for advanced or metastatic non-small-cell lung cancer: application of the ESMO-magnitude of clinical benefit scale v1.1. Acta Oncol. 2021;60(9):1225-1232.

15. von Minckwitz G, Huang CS, Mano MS, et al. Trastuzumab Emtansine for Residual Invasive HER2-Positive Breast Cancer. N Engl J Med. 2019;380(7):617-628.

16. Verma S, Miles D, Gianni L, et al. Trastuzumab emtansine for HER2-positive advanced breast cancer. N Engl J Med. 2012;367(19):1783-1791.

17. Diéras V, Miles D, Verma S, et al. Trastuzumab emtansine versus capecitabine plus lapatinib in patients with previously treated HER2-positive advanced breast cancer (EMILIA): a descriptive analysis of final overall survival results from a randomised, open-label, phase 3 trial. Lancet Oncol. 2017;18(6):732-742.

18. Welslau M, Diéras V, Sohn JH, et al. Patient-reported outcomes from EMILIA, a randomized phase 3 study of trastuzumab emtansine (T-DM1) versus capecitabine and lapatinib in human epidermal growth factor receptor 2-positive locally advanced or metastatic breast cancer. Cancer. 2014;120(5):642-651.

19. Powles T, Rosenberg JE, Sonpavde GP, et al. Enfortumab Vedotin in Previously Treated Advanced Urothelial Carcinoma. N Engl J Med. 2021;384(12):1125-1135.

20. Modi S, Saura C, Yamashita T, et al. Trastuzumab Deruxtecan in Previously Treated HER2-Positive Breast Cancer. N Engl J Med. 2020;382(7):610-621.

For peer review only - http://bmjopen.bmj.com/site/about/guidelines.xhtml

21. Cortés J, Kim SB, Chung WP, et al. Trastuzumab Deruxtecan versus Trastuzumab Emtansine for Breast Cancer. N Engl J Med. 2022;386(12):1143-1154.

22. Hurvitz SA, Hegg R, Chung WP, et al. Trastuzumab deruxtecan versus trastuzumab emtansine in patients with HER2-positive metastatic breast cancer: updated results from DESTINY-Breast03, a randomised, open-label, phase 3 trial. Lancet. 2023;401(10371):105-117.

23. Modi S, Jacot W, Yamashita T, et al. Trastuzumab Deruxtecan in Previously Treated HER2-Low Advanced Breast Cancer. N Engl J Med. 2022;387(1):9-20.

24. Shitara K, Bang YJ, Iwasa S, et al. Trastuzumab Deruxtecan in Previously Treated HER2-Positive Gastric Cancer. N Engl J Med. 2020;382(25):2419-2430.

25. Goto K, Goto Y, Kubo T, et al. Trastuzumab Deruxtecan in Patients With HER2-Mutant Metastatic Non-Small-Cell Lung Cancer: Primary Results From the Randomized, Phase II DESTINY-Lung02 Trial. J Clin Oncol. 2023;JCO2301361.

26. Bardia A, Hurvitz SA, Tolaney SM, et al. Sacituzumab Govitecan in Metastatic Triple-Negative Breast Cancer. N Engl J Med. 2021;384(16):1529-1541.

27. Loibl S, Loirat D, Tolaney SM, et al. Health-related quality of life in the phase III ASCENT trial of sacituzumab govitecan versus standard chemotherapy in metastatic triple-negative breast cancer. Eur J Cancer. 2023;178:23-33.

28. Rugo HS, Bardia A, Marmé F, et al. Sacituzumab Govitecan in Hormone Receptor-Positive/Human Epidermal Growth Factor Receptor 2-Negative Metastatic Breast Cancer. J Clin Oncol. 2022;40(29):3365-3376.

29. Rugo HS, Bardia A, Marmé F, et al. Overall survival with sacituzumab govitecan in hormone receptor-positive and human epidermal growth factor receptor 2-negative metastatic breast cancer (TROPiCS-02): a randomised, open-label, multicentre, phase 3 trial. Lancet. 2023;S0140-6736(23)01245-X.

30. Tagawa ST, Balar AV, Petrylak DP, et al. TROPHY-U-01: A Phase II Open-Label Study of Sacituzumab Govitecan in Patients With Metastatic Urothelial Carcinoma Progressing After Platinum-Based Chemotherapy and Checkpoint Inhibitors. J Clin Oncol. 2021;39(22):2474-2485.

For peer review only - http://bmjopen.bmj.com/site/about/guidelines.xhtml

31. Cognetti DM, Johnson JM, Curry JM, et al. Phase 1/2a, open-label, multicenter study of RM-1929 photoimmunotherapy in patients with locoregional, recurrent head and neck squamous cell carcinoma. Head Neck. 2021;43(12):3875-3887.

32. Peng Z, Liu T, Wei J, et al. Efficacy and safety of a novel anti-HER2 therapeutic antibody RC48 in patients with HER2-overexpressing, locally advanced or metastatic gastric or gastroesophageal junction cancer: a single-arm phase II study. Cancer Commun (Lond). 2021;41(11):1173-1182.

33. Sheng X, Yan X, Wang L, et al. Open-label, Multicenter, Phase II Study of RC48-ADC, a HER2-Targeting Antibody-Drug Conjugate, in Patients with Locally Advanced or Metastatic Urothelial Carcinoma. Clin Cancer Res. 2021;27(1):43-51.

34. Coleman RL, Lorusso D, Gennigens C, et al. Efficacy and safety of tisotumab vedotin in previously treated recurrent or metastatic cervical cancer (innovaTV 204/GOG-3023/ENGOT-cx6): a multicentre, open-label, single-arm, phase 2 study. Lancet Oncol. 2021;22(5):609-619.

35. Matulonis UA, Lorusso D, Oaknin A, et al. Efficacy and Safety of Mirvetuximab Soravtansine in Patients With Platinum-Resistant Ovarian Cancer With High Folate Receptor Alpha Expression: Results From the SORAYA Study. J Clin Oncol. 2023;41(13):2436-2445.

36. George S.L. Response rate as an endpoint in clinical trial. J Natl Cancer Inst. 2007;99:98–99.

37. Chen EY, Joshi SK, Tran A, Prasad V. Estimation of Study Time Reduction Using Surrogate End Points Rather Than Overall Survival in Oncology Clinical Trials. JAMA Intern Med. 2019;179(5):642-647.

38. Gyawali B, Hey SP, Kesselheim AS. Assessment of the clinical benefit of cancer drugs receiving accelerated approval. JAMA Intern Med. 2019;179(7):906-913.

39. Pignatti F, Wilking U, Postmus D, et al. The value of anticancer drugs-a regulatory view. Nat Rev Clin Oncol. 2022;19:207–215.

40. Tibau A, Molto C, Borrell M, et al. Magnitude of Clinical Benefit of Cancer Drugs Approved by the US Food and Drug Administration Based on Single-Arm Trials. JAMA Oncol. 2018;4(11):1610-1611.

For peer review only - http://bmjopen.bmj.com/site/about/guidelines.xhtml

41. Gyawali B, de Vries EGE, Dafni U, et al. Biases in study design, implementation, and data analysis that distort the appraisal of clinical benefit and ESMO-Magnitude of Clinical Benefit Scale (ESMO-MCBS) scoring. ESMO Open. 2021;6(3):100117.
42. Davis C, Naci H, Gurpinar E, Poplavska E, Pinto A, Aggarwal A. Availability of evidence of benefits on overall survival and quality of life of cancer drugs approved by European Medicines Agency: retrospective cohort study of drug approvals 2009-13. BMJ. 2017;359:j4530.
43. Naci H, Davis C, Savović J, et al. Design characteristics, risk of bias, and reporting of randomised controlled trials supporting approvals of cancer drugs by European Medicines Agency, 2014-16: cross sectional analysis. BMJ. 2019;366:l5221.
44. Hilal T, Gonzalez-Velez M, Prasad V. Limitations in Clinical Trials Leading to Anticancer Drug Approvals by the US Food and Drug Administration. JAMA Intern Med. 2020;180(8):1108-1115.
45. Kiesewetter B, Dafni U, de Vries EGE, et al. ESMO-Magnitude of Clinical Benefit Scale for haematological malignancies (ESMO-MCBS:H) version 1.0. Ann Oncol. 2023;34(9):734-771.

For peer review only - http://bmjopen.bmj.com/site/about/guidelines.xhtml

Table 1. Characteristics of the included studies

Drug name	Trial name/ Registry number	Design	Indications	Approval data	Ref
Trastuzumab emtansine	KATHERINE NCT01772472	Phase 3 RCT	HER2-positive early breast cancer after receiving neoadjuvant therapy containing a taxane (with or without anthracycline) and trastuzumab	2019 by FDA 2019 by EMA	15
	EMILIA NCT00829166	Phase 3 RCT	HER2-positive advanced breast cancer second line	2013 by FDA 2013 by EMA	16-18
Enfortumab vedotin	EV-301 NCT03474107	Phase 3 RCT	Locally advanced or metastatic urothelial carcinoma	2019 by FDA 2022 by EMA	19
Trastuzumab deruxtecan	DESTINY-Breast01 NCT03248492	Phase 2 SAT	HER2-positive metastatic breast cancer third line	2019 by FDA 2020 by EMA	20
	DESTINY-Breast03 NCT03529110	Phase 3 RCT	HER2-positive metastatic breast cancer second line	2022 by FDA 2022 by EMA 2023 by NMPA	21, 22
	DESTINY-Breast04 NCT03734029	Phase 3 RCT	HER2-low metastatic breast cancer	2022 by FDA 2022 by EMA 2023 by NMPA	23
	DESTINY-Gastric01 NCT03329690	Phase 2 RCT	HER2-positive advanced gastric or gastroesophageal junctionadenocarcinoma after a prior trastuzumab-based regimen	2021 by FDA 2022 by EMA	24
	DESTINY-Lung02 NCT04644237	Phase 2 SAT	Metastatic HER2-mutant NSCLC second line	2022 by FDA	25
Sacituzumab govitecan	ASCENT NCT02574455	Phase 3 RCT	Relapsed or refractory metastatic triple-negative breast cancer	2020 by FDA 2021 by EMA 2022 by NMPA	26, 27
	TROPiCS-02 NCT03901339	Phase 3 RCT	Endocrine-resistant, chemotherapy-treated HR+/HER2- locally recurrent inoperable or metastatic breast cancer	2023 by FDA 2023 by EMA	28, 29
	TROPHY-U-01 NCT03547973	Phase 2 RCT	Metastatic urothelial carcinoma	2021 by FDA	30
Cetuximab saratolacan	- NCT02422979	Phase 2 SAT	Recurrent head and neck squamous cell carcinoma	2020 by PMDA	31
Disitamab vedotin	- NCT03556345	Phase 2 SAT	HER2-overexpressing, locally advanced or metastatic gastric or gastroesophageal junction cancer	2021 by NMPA	32
	-	Phase 2 SAT	HER2+ locally advanced or metastatic urothelial carcinoma	2022 by NMPA	33

For peer review only - http://bmjopen.bmj.com/site/about/guidelines.xhtml

	NCT03507166				
Tisotumab vedotin	innovaTV 204 NCT03438396	Phase 2 SAT	Recurrent or metastatic cervical cancer	2021 by FDA	34
Mirvetuximab soravtansine	SORAYA NCT04296890	Phase 2 SAT	FRa-high, platinum-resistant epithelial ovarian cancer	2022 by FDA	35

EMA, European Medicines Agency; FDA, Food and Drug Administration; NMDA, China National Medical Products Administration; PMDA, Japanese Pharmaceuticals and Medical Devices Agency; RCT, randomized control trial; SAT, single-arm trial.

For peer review only

For peer review only - http://bmjopen.bmj.com/site/about/guidelines.xhtml

Table 2. Clinical benefit according to ESMO-MCBS v1.1 and concordance rate between ESMO Framework in this study and ESMO-MCBS scorecards

Trial name	Intervention group	PO	PO ctrl group	PO gain	PO HR (95% CI)	Adjustments	ESMO-MCBS v1.1	Scorecards
KATHERINE	T-DM1	iDFS	3-year: 77.0%	3-year: 11.3%	0.50 (0.39-0.64)	NA	A (form 1)	A
EMILIA	T-DM1	PFS and OS	25.9 mo	4.0 mo	0.75 (0.64-0.88)	Improved HRQoL	3 (form 2a)	4
EV-301	EV	OS	8.97 mo	3.91 mo	0.70 (0.56-0.89)	NA	4 (form 2a)	4
DESTINY-Breast01	DS-8201	ORR	-	-	-	NA	2 (form 3)	2
DESTINY-Breast03	DS-8201	PFS (OS improved)	2-year: 69.9%	2-year: 7.5%	0.64 (0.47-0.87)	NA	3 (form 2a)	4
DESTINY-Breast04	DS-8201	PFS (OS improved)	16.8 mo	6.6 mo	0.64 (0.49–0.84)	NA	4 (form 2a)	4
DESTINY-Gastric01	DS-8201	ORR	14%	37%	-	NA	2 (form 2c)	4
DESTINY-Lung02	DS-8201	ORR	-	-	-	NA	3 (form 3)	NA
ASCENT	SG	PFS (OS improved)	6.9 mo	4.9 mo	0.51 (0.41-0.62)	Increased toxicity/ Improved HRQoL	4 (form 2a)	4
TROPiCS-02	SG	PFS (OS improved)	11.2 mo	3.2 mo	0.79 (0.65-0.96)	NA	1 (form 2a)	3
TROPHY-U-01	SG	ORR	-	-	-	NA	3 (form 3)	NA
NCT02422979	CS	ORR	-	-	-	NA	2 (form 3)	NA
NCT03556345	DV	ORR	-	-	-	NA	2 (form 3)	NA
NCT03507166	DV	ORR	-	-	-	NA	3 (form 3)	NA
innovaTV 204	TV	ORR	-	-	-	NA	2 (form 3)	2
SORAYA	MS	ORR	-	-	-	NA	2 (form 3)	2

CS, cetuximab saratolacan; DFS, disease-free survival; DV, disitamab vedotin; EV, enfortumab vedotin; MS, mirvetuximab soravtansine ORR, objective response rate; OS, overall survival; PFS, progression-free survival; PO, primary outcome; SG, sacituzumab govitecan; T-DM1, trastuzumab emtansine; TV, tisotumab vedotin.

For peer review only - http://bmjopen.bmj.com/site/about/guidelines.xhtml

Supplemental Table S1 Search strategy for studies on ADCs in solid tumours

Database	Search Period	Search Terms
Pubmed	2000 to 18 October 2023	1. antibody-drug conjugates<br>2. solid cancer<br>3. approve<br>4. clinical trials<br>5. quality of life<br>6. follow-up survival<br>7. updated overall survival

For peer review only

PRISMA 2020 Checklist

Section and Topic	Item #	Checklist item	Location where item is reported
**TITLE**			
Title	1	Identify the report as a systematic review.	Page 1
**ABSTRACT**			
Abstract	2	See the PRISMA 2020 for Abstracts checklist.	Page 2
**INTRODUCTION**			
Rationale	3	Describe the rationale for the review in the context of existing knowledge.	Page 4
Objectives	4	Provide an explicit statement of the objective(s) or question(s) the review addresses.	Page 4
**METHODS**			
Eligibility criteria	5	Specify the inclusion and exclusion criteria for the review and how studies were grouped for the syntheses.	Page 5
Information sources	6	Specify all databases, registers, websites, organisations, reference lists and other sources searched or consulted to identify studies. Specify the date when each source was last searched or consulted.	Page 5
Search strategy	7	Present the full search strategies for all databases, registers and websites, including any filters and limits used.	Page 5 Supplemental Table S1
Selection process	8	Specify the methods used to decide whether a study met the inclusion criteria of the review, including how many reviewers screened each record and each report retrieved, whether they worked independently, and if applicable, details of automation tools used in the process.	Page 5
Data collection process	9	Specify the methods used to collect data from reports, including how many reviewers collected data from each report, whether they worked independently, any processes for obtaining or confirming data from study investigators, and if applicable, details of automation tools used in the process.	Page 5
Data items	10a	List and define all outcomes for which data were sought. Specify whether all results that were compatible with each outcome domain in each study were sought (e.g. for all measures, time points, analyses), and if not, the methods used to decide which results to collect.	Page 5
	10b	List and define all other variables for which data were sought (e.g. participant and intervention characteristics, funding sources). Describe any assumptions made about any missing or unclear information.	Page 5
Study risk of bias assessment	11	Specify the methods used to assess risk of bias in the included studies, including details of the tool(s) used, how many reviewers assessed each study and whether they worked independently, and if applicable, details of automation tools used in the process.	Page 5
Effect measures	12	Specify for each outcome the effect measure(s) (e.g. risk ratio, mean difference) used in the synthesis or presentation of results.	Page 5-6
Synthesis methods	13a	Describe the processes used to decide which studies were eligible for each synthesis (e.g. tabulating the study intervention characteristics and comparing against the planned groups for each synthesis (item #5)).	NA
	13b	Describe any methods required to prepare the data for presentation or synthesis, such as handling of missing summary statistics, or data conversions.	NA
	13c	Describe any methods used to tabulate or visually display results of individual studies and syntheses.	NA
	13d	Describe any methods used to synthesize results and provide a rationale for the choice(s). If meta-analysis was performed, describe the model(s), method(s) to identify the presence and extent of statistical heterogeneity, and software package(s) used.	NA
	13e	Describe any methods used to explore possible causes of heterogeneity among study results (e.g. subgroup analysis, meta-regression).	NA
	13f	Describe any sensitivity analyses conducted to assess robustness of the synthesized results.	NA
Reporting bias	14	Describe any methods used to assess risk of bias due to missing results in a synthesis (arising from reporting biases).	NA

For peer review only - http://bmjopen.bmj.com/site/about/guidelines.xhtml

PRISMA 2020 Checklist

Section and Topic	Item #	Checklist item	Location where item is reported
assessment			
Certainty assessment	15	Describe any methods used to assess certainty (or confidence) in the body of evidence for an outcome.	NA
**RESULTS**			
Study selection	16a	Describe the results of the search and selection process, from the number of records identified in the search to the number of studies included in the review, ideally using a flow diagram.	Page 6
	16b	Cite studies that might appear to meet the inclusion criteria, but which were excluded, and explain why they were excluded.	Page 6
Study characteristics	17	Cite each included study and present its characteristics.	Page 6, Table 1
Risk of bias in studies	18	Present assessments of risk of bias for each included study.	NA
Results of individual studies	19	For all outcomes, present, for each study: (a) summary statistics for each group (where appropriate) and (b) an effect estimate and its precision (e.g. confidence/credible interval), ideally using structured tables or plots.	Page 6, Table 1
Results of syntheses	20a	For each synthesis, briefly summarise the characteristics and risk of bias among contributing studies.	NA
	20b	Present results of all statistical syntheses conducted. If meta-analysis was done, present for each the summary estimate and its precision (e.g. confidence/credible interval) and measures of statistical heterogeneity. If comparing groups, describe the direction of the effect.	NA
	20c	Present results of all investigations of possible causes of heterogeneity among study results.	NA
	20d	Present results of all sensitivity analyses conducted to assess the robustness of the synthesized results.	NA
Reporting biases	21	Present assessments of risk of bias due to missing results (arising from reporting biases) for each synthesis assessed.	NA
Certainty of evidence	22	Present assessments of certainty (or confidence) in the body of evidence for each outcome assessed.	NA
**DISCUSSION**			
Discussion	23a	Provide a general interpretation of the results in the context of other evidence.	Page 7
	23b	Discuss any limitations of the evidence included in the review.	Page 10
	23c	Discuss any limitations of the review processes used.	Page 10
	23d	Discuss implications of the results for practice, policy, and future research.	Page 9-10
**OTHER INFORMATION**			
Registration and protocol	24a	Provide registration information for the review, including register name and registration number, or state that the review was not registered.	Page 5
	24b	Indicate where the review protocol can be accessed, or state that a protocol was not prepared.	Page 5
	24c	Describe and explain any amendments to information provided at registration or in the protocol.	NA
Support	25	Describe sources of financial or non-financial support for the review, and the role of the funders or sponsors in the review.	Page 11
Competing interests	26	Declare any competing interests of review authors.	Page 11
Availability of data, code and	27	Report which of the following are publicly available and where they can be found: template data collection forms; data extracted from included studies; data used for all analyses; analytic code; any other materials used in the review.	Page 11

For peer review only - http://bmjopen.bmj.com/site/about/guidelines.xhtml

PRISMA 2020 Checklist

Section and Topic	Item #	Checklist item	Location where item is reported
other materials			

From: Page MJ, McKenzie JE, Bossuyt PM, Boutron I, Hoffmann TC, Mulrow CD, et al. The PRISMA 2020 statement: an updated guideline for reporting systematic reviews. BMJ 2021;372:n71. doi: 10.1136/bmj.n71

For more information, visit: http://www.prisma-statement.org/

For peer review only
